# PW1/Peg3 expression regulates key properties that determine mesoangioblast stem cell competence

**DOI:** 10.1038/ncomms7364

**Published:** 2015-03-09

**Authors:** Chiara Bonfanti, Giuliana Rossi, Francesco Saverio Tedesco, Monica Giannotta, Sara Benedetti, Rossana Tonlorenzi, Stefania Antonini, Giovanna Marazzi, Elisabetta Dejana, David Sassoon, Giulio Cossu, Graziella Messina

**Affiliations:** 1 Department of BioSciences, University of Milan, 20133 Milan, Italy; 2 Department of Cell and Developmental Biology, University College London, 21 University Street, London WC1E 6DE, UK; 3 IFOM, FIRC Institute of Molecular Oncology, 20139 Milan, Italy; 4 Institute of Child Health, University College London, 30 Guilford Street, London WC1N 1EH, UK; 5 San Raffaele Hospital, Division of Neuroscience-INSPE, via Olgettina 58, 20132 Milan, Italy; 6 Stem Cells and Regenerative Medicine, ICAN UMRS 1166 Inserm/Sorbonne University (UPMC ParisVI), 75634 Paris, France; 7 Institute of Inflammation and Repair, University of Manchester, Oxford Road, Manchester M13 9PL, UK

## Abstract

Mesoangioblasts are vessel-associated progenitor cells that show therapeutic promise for the treatment of muscular dystrophy. Mesoangioblasts have the ability to undergo skeletal muscle differentiation and cross the blood vessel wall regardless of the developmental stage at which they are isolated. Here we show that PW1/Peg3 is expressed at high levels in mesoangioblasts obtained from mouse, dog and human tissues and its level of expression correlates with their myogenic competence. Silencing PW1/Peg3 markedly inhibits myogenic potential of mesoangioblasts *in vitro* through MyoD degradation. Moreover, lack of PW1/Peg3 abrogates mesoangioblast ability to cross the vessel wall and to engraft into damaged myofibres through the modulation of the junctional adhesion molecule-A. We conclude that PW1/Peg3 function is essential for conferring proper mesoangioblast competence and that the determination of PW1/Peg3 levels in human mesoangioblasts may serve as a biomarker to identify the best donor populations for therapeutic application in muscular dystrophies.

## Abstract (web summary)

Mesoangioblasts are mesodermal stem cells with a therapeutic potential for treatment of muscular dystrophy due to their ability to differentiate into skeletal muscle. This study shows that the PW1/Peg3 protein is crucial for mesoangioblast myogenic and migratory potency and is a therapeutically relevant biomarker.

## Graphical abstract

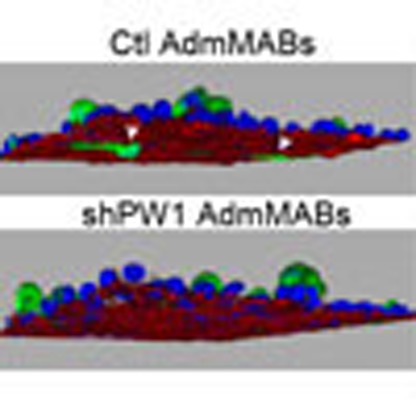

Mesoangioblasts (MABs) are blood vessel-associated progenitor cells that can differentiate into mesoderm cell types, including skeletal muscle^1^. When delivered through the arterial circulation, MABs cross the blood vessel wall and participate in skeletal muscle regeneration leading to an amelioration of muscular dystrophies in different pre-clinical animal models: the *α-sarcoglican-null* mouse, which models the limb-girdle muscular dystrophy, the AJ mouse model of dysferlinopathy, the *mdx* mouse for Duchenne muscular dystrophy (DMD)^2^,^3^,^4^,^5^ and the golden retriever muscular dystrophy dog^6^. The ability of MABs to cross the vessel wall confers an advantage as therapeutic donor stem cells as compared with satellite cells and myoblasts that need to be delivered directly into the muscle tissue to properly engraft^7^,^8^. Cells with MAB-like properties have been isolated from human adult skeletal muscle pericytes^9^ and expanded under clinical-grade conditions, providing the basis for a Phase I/II clinical trial for Duchenne muscular dystrophy (EudraCT no. 2011-000176-33; Cossu *et al*. in preparation).

Since the initial identification of MABs in mouse embryos^1^, all MABs thus far isolated from different species (mouse, dog and human) and developmental stages have been shown to contribute to muscle regeneration *in vivo*. Although alkaline phosphatase expression can be used to identify adult human MABs in skeletal muscle, no single unequivocal marker has been identified that denotes MAB identity across species^1^,^4^,^6^,^9^. As a result, MABs are defined predominantly by their isolation method and functional properties. A cross-analysis of independent microarrays performed on different human and murine MAB populations revealed that PW1 is expressed in MABs regardless of species origin and age of isolation^10^. PW1, also identified as Peg3 (Paternally expressed gene3) was initially isolated following a screening to identify factors involved in skeletal muscle commitment as well as in a screen for parentally imprinted genes^11^. PW1/Peg3 (hereafter referred to as PW1) is expressed at high levels upon gastrulation and is downregulated during fetal and perinatal development^11^,^12^. In postnatal skeletal muscle, PW1 expression is restricted to muscle satellite cells and to a subpopulation of interstitial cells, which display myogenic potential^13^,^14^,^15^. Moreover, PW1 expression identifies multiple adult stem and progenitor cell populations from a wide array of adult tissues including skin, gut, blood and central nervous system^16^. PW1 encodes a zinc-finger protein that has been shown to participate in inflammatory- and p53-mediated cell stress pathways^17^,^18^ as well as a mediator of β-catenin stability in the Wnt signalling pathway^19^. PW1 has also been shown to function as a transcription factor with a DNA-binding motif regulating an array of genes involved in metabolic homeostasis^20^.

We report here that silencing *PW1* in a polyclonal population of murine MABs abrogates their capacity to differentiate into skeletal muscle and inhibits their ability to cross the vessel wall and therefore migrate towards damaged muscle. We observed that PW1 controls MAB muscle differentiation by stabilizing MyoD via regulation of cyclinE levels and regulates engraftment efficiency by modulating the expression of molecules responsible for trans-vessel migration, including the tight junction molecule JAM-A. Consistent with these observations, we found that levels of PW1 expression correlate with the myogenic and migratory capacities of both murine- and human-derived MABs, indicating that PW1 expression levels can be used to screen and identify competent MABs before their use in cell therapy.

## Results

### PW1 characterizes MABs and their myogenic competence

**Figure 1:**
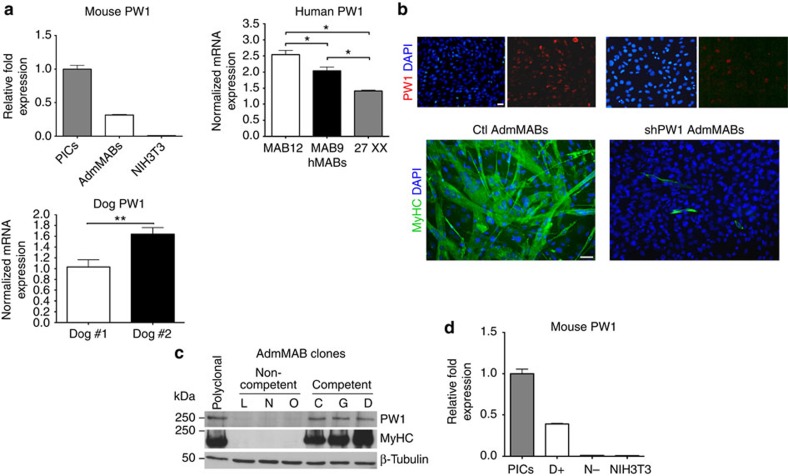
Silencing of *PW1* interferes with mesoangioblasts (MABs) muscle differentiation. (**a**) PW1 expression by qRT–PCR on different populations of mouse adult (AdmMABs), human and canine MABs. Values are plotted as relative messenger RNA (mRNA) expression and normalized to GAPDH levels. For the AdmMABs, values are expressed as fold expression relative to subpopulation of interstitial cells (PICs; =1). Each assay was performed in triplicate. Data are represented as means±s.d. *P<0.05, ns, not significant one-way unpaired *t* Test. (**b**) Immunofluorescence analysis for PW1 (red) and for the expression of all sarcomeric myosins (MyHC, green) on Ctl and shPW1 AdmMAB growing cells upon 5 days in differentiation medium. DAPI was used to stain nuclei. Scale bar represents 100 and 50 μm. (**c**) Western blot analysis of MyHC and PW1 expression on six different clones of AdmMABs isolated and selected for the different myogenic potency. Clones have been divided in competent (C, G, D) and non-competent (L, N, O) on the basis of their myogenic property. β-Tubulin was used to normalize the amount of loaded proteins. Polyclonal AdmMABs were used as a positive control. (**d**) PW1 expression by qRT–PCR on representative clones of competent (D+) and non-competent AdmMABs (N−). Values are normalized to GAPDH levels and expressed as fold expression relative to PICs (=1). NIH3T3 fibroblasts were used as negative control.

We previously generated independent microarray gene expression profiles from MABs isolated from mouse and human donors with the aim to select common markers^10^. Here we focused upon PW1 since it has been shown to identify adult stem and progenitor cell populations in different tissues, including skeletal muscle^13^,^16^. From these arrays, PW1 was found to be expressed in MABs regardless of species and age^9^,^10^. PW1 expression in mouse, dog and human MABs was also confirmed by quantitative PCR with reverse transcription (qRT–PCR) (Fig. 1a). Although PW1 provides a tool as a cross-species marker, we wished to understand its role in MABs. We therefore silenced PW1 expression in a polyclonal population of adult mouse MABs (AdmMABs) by using a lentiviral vector expressing a short hairpin RNA sequence for PW1 (shPW1). We chose AdmMABs since, at variance with embryonic mMABs, they spontaneously differentiate in culture without the need of a co-culture with myoblasts^4^. As shown in Fig. 1b, silencing of PW1 led to a marked reduction of skeletal muscle differentiation. We established 37 clones from the parental population and assessed their myogenic competence and levels of PW1 expression. Six clones were chosen on the basis of their different levels of myogenic competence. We observed that clones displaying high levels of myogenic competence (competent clones C, G and D) expressed high levels of PW1, whereas clones with low or no myogenic capacity (non-competent clones L, N and O) displayed undetectable levels of PW1 (Fig. 1c,d, Supplementary Fig. 1). We then tested the effects of PW1 silencing on the well-characterized embryonic mouse-derived MAB clone, D16 (refs 1, 2). As seen with AdmMABs, we observed a comparable inhibition of myogenesis following PW1 silencing (Supplementary Fig. 2a,b).

We had previously demonstrated that myogenic competence requires Pax3 expression in embryonic-derived mMABs^21^. In contrast, silencing of Pax3 in AdmMABs does not affect myogenic competence, revealing a developmental stage-specific requirement for Pax3 in MABs (Supplementary Fig. 3a,b). In agreement with this observation, PW1 expression was undetectable in Pax3 null embryonic mMABs (Supplementary Fig. 3c,d) but present at normal levels in Pax3 silenced AdmMABs (Supplementary Fig. 3b). These data demonstrate that PW1 expression is tightly linked with embryonic and adult mMAB myogenic competence, whereas Pax3 is required uniquely for embryonic myogenic mMAB competence.

### PW1 is required for MAB differentiation via MyoD

**Figure 2:**
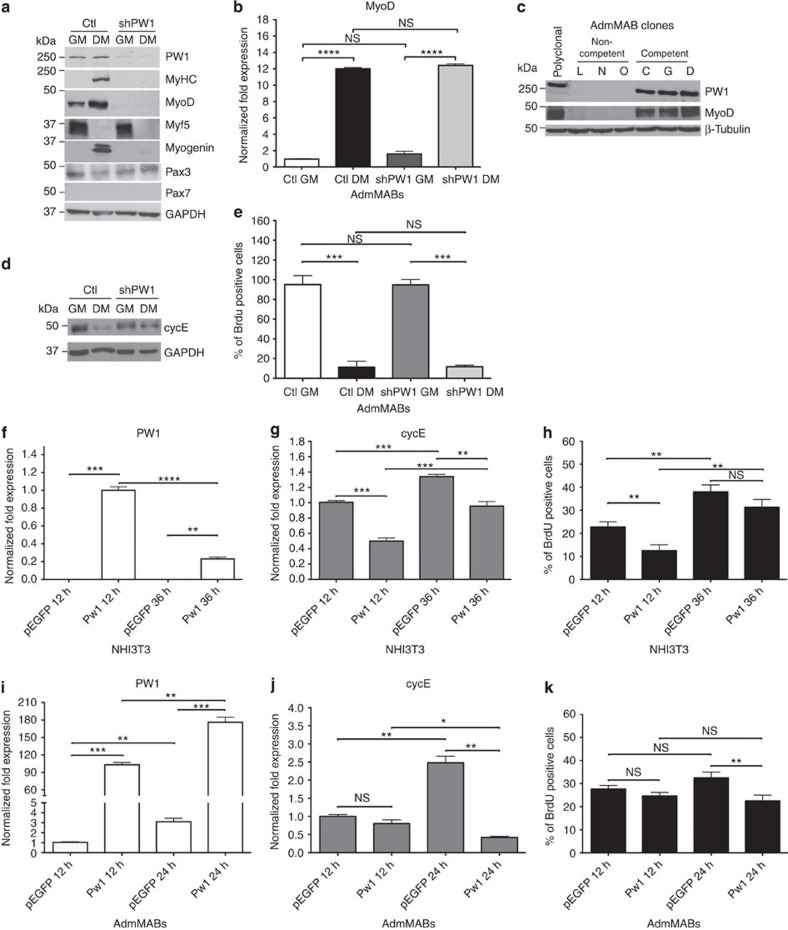
PW1 is necessary for MAB myogenic competence and negatively regulates cyclinE expression. (**a**) Western blot for PW1 and myogenic markers expressed by Ctl and shPW1 AdmMABs. GAPDH was used to normalize. GM, growing medium; DM, differentiation medium. (**b**) MyoD expression by qRT–PCR on Ctl and shPW1 AdmMABs in GM and DM. Values are plotted as relative fold expression and normalized to GAPDH expression. Each assay was performed in triplicate. Data are represented as means ± s.d. ****P<0.0001, NS, not significant one-way unpaired *t*-test. (**c**) Western blot for PW1 and MyoD expression on non-competent (L, N, O) and competent (C, G, D) AdmMAB clones. β-Tubulin was used to normalize. (**d**) Western blot for cycE expression in Ctl versus shPW1 AdmMABs in growing and differentiating conditions. GAPDH was used to normalize. (**e**) 5′-bromo-deoxyuridine (BrdU) incorporation assay for Ctl and shPW1 AdmMABs. Each assay was performed in triplicate. Data are represented as means ± s.d. ***P<0.0005, NS, not significant one-way unpaired *t*-test. (**f**,**g**) Real time analysis showing PW1 (**f**) and cycE (**g**) levels in NIH3T3 cells transfected with plasmid expressing PW1 (PW1) or an empty vector, as control (pEGFP) after 12 (12 h) and 36 h (36 h) from the transfection. Values are plotted as relative fold expression and normalized to GAPDH expression. Each assay was performed in triplicate. (**h**) BrdU incorporation assay on transfected NIH3T3 mouse fibroblasts. (**i**,**j**) Real time analysis showing PW1 (**i**) and CycE (**j**) levels on AdmMABs. Cells have been transiently transfected with plasmid expressing PW1 (PW1) and an empty vector, as control (pEGFP). Cells have been analysed after 12 (12 h) and 24 h (24 h) from the transfection. Values are plotted as relative fold expression and normalized to GAPDH expression. Each assay was performed in triplicate. (**k**) BrdU incorporation assay on transfected AdmMABs by incubating with 50 μM BrdU for 1 h, just before being stopped for the following qRT–PCR analysis. Each assay was performed in triplicate. All data are represented as means ± s.d. *P<0.05, **P<0.005, ***P<0.0005, ****P<0.0001, NS, not significant, one-way unpaired *t*-test.

To define the molecular pathway through which PW1 regulates MAB myogenic competence, we examined key regulators of the myogenic programme in shPW1 AdmMABs versus control cells (Ctl). We observed a marked decrease in MyoD protein in the absence of PW1 expression (Fig. 2a). In contrast, MyoD transcripts were present and upregulated during differentiation regardless of short hairpin RNA treatment (Fig. 2b). Consistent with these observations, we observed that MyoD protein is absent in non-competent AdmMAB clones, which spontaneously lack PW1 (Fig. 2c). It has been previously demonstrated that activation of Cdk-dependent phosphorylation of MyoD leads to its rapid degradation and moreover, direct phosphorylation of MyoD on Ser200 by the complex cycE–Cdk2 (cyclinE/Cyclin dependent kinase2) regulates its stability^22^,^23^,^24^. We therefore measured cycE protein levels in Ctl versus shPW1 AdmMABs during myogenic differentiation. Cells were cultured in growth conditions and then shifted to differentiation medium (DM) for 5 days. We observed that Ctl AdmMABs downregulated cycE in DM, as expected, whereas cycE levels remained high in the shPW1 AdmMABs (Fig. 2d). Even though levels of cycE remained high following PW1 silencing, we noted that they normally exit from cell cycle as demonstrated by 5′-bromo-deoxyuridine (BrdU) labelling (Fig. 2e). We therefore investigated the possibility of cycE regulation by PW1. Since NIH3T3 fibroblasts do not express PW1 (ref. 17), proliferating NIH3T3 cells were transiently transfected with a plasmid expressing PW1 or with an pEGFP Ctl vector: cycE expression was evaluated by qRT–PCR after 12 and 36 h from transfection. We observed a marked decrease in cycE expression in response to PW1 (Fig. 2f,g). Moreover, we observed that in fibroblasts that do not undergo a cell cycle-dependent programme of lineage commitment, the proliferation rate was decreased two-fold following PW1 forced expression (Fig. 2h), consistent with previous results^18^. We next verified if myogenic competent AdmMABs also displayed PW1-mediated inhibition of cycE and subsequent regulation of MyoD activity. Therefore, we transiently transfected AdmMABs with either PW1 or with the empty Ctl vector pEGFP (Fig. 2i). After 24 h from PW1 transfection, we observed that cycE was markedly downregulated (Fig. 2j). In this case, the proliferation rate of AdmMABs overexpressing PW1 was less altered than what was observed for the PW1-NIH3T3 cells (Fig. 2k).

**Figure 3:**
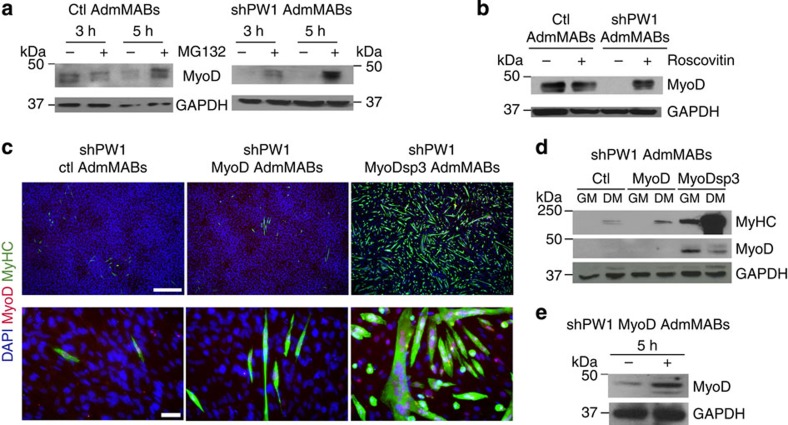
Silencing of PW1 leads to MyoD degradation via cycE/Cdk2-proteasome-dependent pathway. (**a**) Western blot analysis of MyoD accumulation in Ctl and shPW1 AdmMABs following treatments with 50 μM of the proteasome inhibitor MG132, for 3 and 5 h and (**b**) 5 μM of the cdk2 inhibitor, Roscovitin for 5 h.The +refers to MG132- or Roscovitin-treated cells, whereas − refers to only DMSO-treated cells. GAPDH was used to normalize the amount of loaded proteins. (**c**) Immunofluorescence staining for myosin heavy chain (MyHC, green), MyoD (red) and nuclei (DAPI) on shPW1 AdmMABs transduced with retroviral vector expressing *wt* MyoD (shPW1 MyoD AdmMABs), mutated MyoD (shPW1 MyoDsp3 AdmMABs) and empty control vector (shPW1 Ctl AdmMABs). Scale bars, 500 and 75 μm. (**d**) Western blot analysis of the experiment described in **c**: MyoD and myosin heavy chain (MyHC) expression were checked in proliferating (GM) and differentiated (DM) shPW1 AdmMABs transduced with retrovirus expressing wt MyoD (MyoD), mutated MyoD (MyoDsp3) and empty control vector (Ctl). GAPDH was used to normalize the amount of loaded proteins. (**e**) MyoD expression, evaluated by western blot, in shPW1 AdmMABs stably transduced with the retrovirus expressing the wt MyoD (shPW1 MyoD) and treated for 5 h with the proteasome inhibitor MG132. The +refers to MG132-treated cells, whereas − refers to only DMSO-treated cells. GAPDH was used to normalize the amount of loaded proteins.

The cycE-dependent degradation pathway of MyoD is due to proteasome activity^22^,^23^,^24^. We therefore interfered with this pathway in Ctl and shPW1 AdmMabs at two different levels: at the level of the proteasome activity by using the proteasome inhibitor MG132 and at the level of cycE/Cdk2 activity by using Roscovitin (a Cdk2 inhibitor). As shown in Fig. 3a,b, inhibition of proteasome or cycE/Cdk2 function led to MyoD protein accumulation in shPW1 AdmMABs. To confirm these observations, we tested whether ectopically expressed MyoD would rescue differentiation in the shPW1 AdmMabs using both wt or a phosphorylation-resistant form of MyoD (MyoD, MyoDsp3)^25^. Ctl, MyoD or MyoDsp3 transduced-shPW1 AdmMABs were induced to differentiate and we observed that only MyoDsp3 forced expression led to a complete rescue of terminal myogenic differentiation (Fig. 3c,d). Notably, we additionally performed the same experiments in non-competent clones and obtained the same results (Supplementary Fig. 4). We reasoned that MyoD forced expression did not rescue the myogenic programme in shPW1 AdmMabs due to a high rate of MyoD degradation (Fig. 3d). We confirmed this hypothesis by treating these cells with MG132 and observed that MyoD protein properly accumulated when the proteasome pathway was blocked, suggesting that in the absence of PW1 expression, the rate of MyoD degradation is high (Fig. 3e).

### AdmMABs require PW1 to cross vessels and engraft muscles

**Figure 4:**
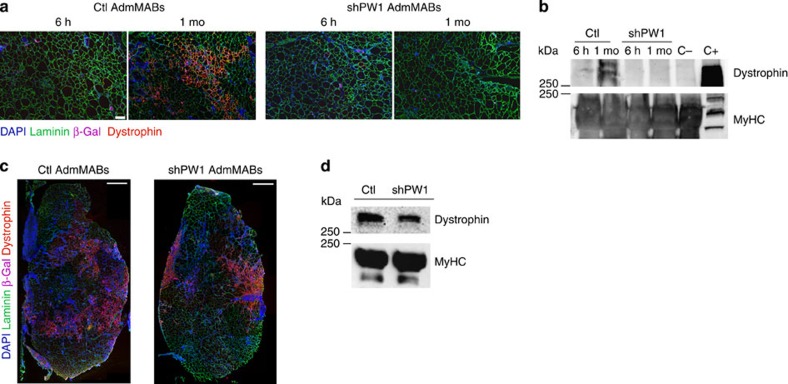
PW1-silenced MABs do not rescue the dystrophic phenotype after intra-arterial transplantation in scid-*mdx* mouse. (**a**) Immunofluorescence staining for laminin (green), dystrophin (red), β-Gal (pink) and nuclei (DAPI, blue) on serial transverse sections of gastrocnemius muscle, 6 h and 1 month after intra-femoral artery injection of n-LacZ Ctl and shPW1 adult murine MABs (AdmMABs) into scid-*mdx* mice. Scale bar, 100 μm. *n*=4 for each group. (**b**) Western blot analysis of dystrophin expression in transplanted scid-*mdx* muscles, 6 h and 1 month (1 mo) after cell transplantation. C+ is a wt muscle, used as a positive control for Dystrophin expression; C− is an *mdx*-not transplanted muscle, representing the negative control for dystrophin expression. The expression of all the sarcomeric myosins, MyHC, was used to normalize the amount of loaded proteins. The C+ was loaded 10 times less to avoid Ab titration and photo bleaching. (**c**) Immunofluorescence staining for laminin (green), dystrophin (red), β-Gal (pink) and nuclei (DAPI, blue) on the transplanted tibialis anterior muscle, 1 month after intra-muscular injection of n-LacZ Ctl and shPW1 adult murine MABs (AdmMABs) into scid-*mdx* mice. *n*=4 for each group. Scale bar, 500 μm. (**d**) Western blot analysis of dystrophin expression in transplanted scid-*mdx* muscles, 1 month after cell transplantation. The expression of all the sarcomeric myosins, MyHC, was used to normalize the amount of loaded proteins.

**Figure 5:**
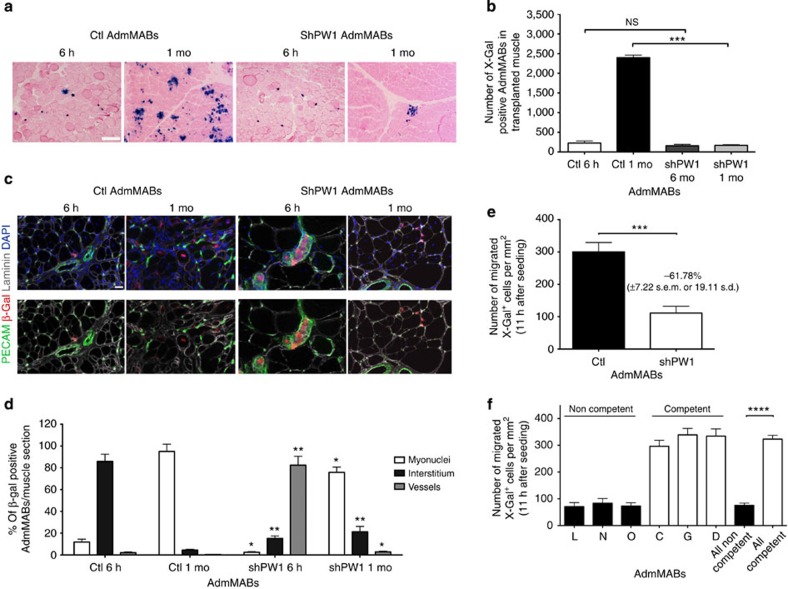
Silencing of PW1 impairs mesoangioblast ability to cross the vessel wall. (**a**) Eosin and X-Gal staining on serial transverse sections of gastrocnemius muscle of transplanted scid-*mdx* mice, 6 h and 1 month (mo) after intra-arterial injection of n-LacZ Ctl and shPW1 cells. X-Gal was used to identify transplanted n-LacZ MABs. Scale bar, 200 μm. This observation has been quantified in the graph (**b**) Values are plotted as total number of X-Gal positive MABs in transplanted grastrocnemius muscles (*n*=4). Data are means (±s.d.) for each group. ***P<0.0005, NS, not significant, unpaired one-way *t*-test. (**c**) Immunofluorescence staining for laminin (grey), β-Gal (red), PECAM (green) and nuclei (DAPI) has been performed on the serial transverse sections of the transplanted muscle. Scale bar represents 25 μm. The result from this analysis has been quantified in the graph (**d**). Values are plotted as % of β-Gal positive MABs per muscle section (both Ctl and shPW1 AdmMABs) associated to vessel (PECAM positive), inside or outside (interstitium) the myofibres (by using laminin as reference). Statistical analysis has been performed comparing, for each time point, the shPW1 AdmMABs column with the respective Ctl column for the different markers. Each assay was performed in triplicate. All data are represented as means ± s.d.*P<0.5 **P<0.005, unpaired one-way *t*-test. (**e**,**f**) The H5V Endothelial cells were seeded on gelatin-coated filters. Ctl (AdmMABs) and shPW1 MABs (shPW1 AdmMABs) (**e**) or competent (C, G and D) and non-competent (L, N and O) AdmMAB clones (**f**) were added to the upper chamber and allowed to migrate for 11 h. Migrated MABs on the lower side of the filters (X-Gal blue nuclei) were fixed and counted. Quantification of migrated AdmMABs per area is shown. Data are means (±s.d.) from five independent experiments, each of these was run in triplicate. ***P<0.0005, ****P<0.0001, unpaired one-way *t*-test.

To assess whether the absence of PW1 impairs myogenic differentiation *in vivo*, we injected Ctl or shPW1 AdmMABs, previously transduced with a lentiviral vector carrying *nLacZ* (to improve detection), into the femoral artery of dystrophic immunodeficient scid-*mdx* mice (to avoid immune rejection against the β-galactosidase). Mice were killed at both 6 h and 1 month after injection. As expected, Ctl AdmMabs gave rise to dystrophin expressing fibres 1 month after injection. In contrast, no dystrophin expression was observed following injection of shPW1 AdmMABs (Fig. 4a,b). Notably, at 6 h post injection, we observed that the shPW1 AdmMABs were primarily excluded from the myofibres and associated with the vessels suggesting a possible impairment of MAB trans-vessel migration in absence of PW1 (Fig. 5c,d). We therefore performed intra-muscular injection of n-LacZ Ctl or shPW1 AdmMABs in the tibialis anterior of the scid-*mdx* mice. We observed that 1 month following intra-muscular cell transplantation, shPW1 AdmMABs restored dystrophin expression in the injected area of scid-*mdx* mice (Fig. 4c,d). This evidence suggests that the shPW1 MAB myogenic competent correction by fusion with the resident regenerating myofibers, although notably in absence of PW1, AdmMABs migrate less, as shown by clustered dystrophin expressing myofibres (Fig. 4c,d). This result most likely reflects the shPW1 AdmMAB inability to properly differentiate and express dystrophin following systemic delivery into scid-*mdx* mice was primarily due to shPW1 AdmMAB impairment to cross the vessel wall.

We observed that while the number of β-Gal+ cells in the muscle was comparable between Ctl and shPW1 cells 6 h following intra-femoral artery injection, only few shPW1 cells were detected in the muscle 1 month after injection (Fig. 5a,b). To identify the tissue distribution of the injected AdmMABs, we stained muscle sections with PECAM (blood vessels), laminin (fiber basal lamina) and β-Gal (transplanted MABs; Fig. 5c). We observed that the majority of Ctl AdmMABs were localized in the interstitium after 6 h, whereas the shPW1 AdmMABs were still within the blood vessels and had not migrated inside the muscle insterstitium (Fig. 5c,d). After 1 month, β-Gal nuclear labelling was primarily observed in differentiated myofibres following Ctl AdmMAB injection, whereas very few labelled nuclei were associated with myofibres in the shPW1 AdmMABs-injected mice (Fig. 5c,d).

These observations led us to propose that AdmMABs are not able to cross the endothelium in the absence of PW1. To address this hypothesis, we created an *in vitro* environment, which allows AdmMABs to face and cross endothelium-coated filters, partly resembling an *in vivo* context. We exploited the murine endothelial cell line, H5V for a transmigration assay. H5V cells were grown to confluence as a monolayer on gelatin-coated filters. β-Gal-labelled Ctl and shPW1 AdmMABs were tested for their capacity to transmigrate through the monolayer. These experiments revealed a twofold decrease in the number of AdmMABs crossing the monolayer in the absence of PW1 (Fig. 5e). We further noted that the ability to cross the monolayer in this assay was present only in the competent AdmMAB clones (Fig. 5f).

**Figure 6:**
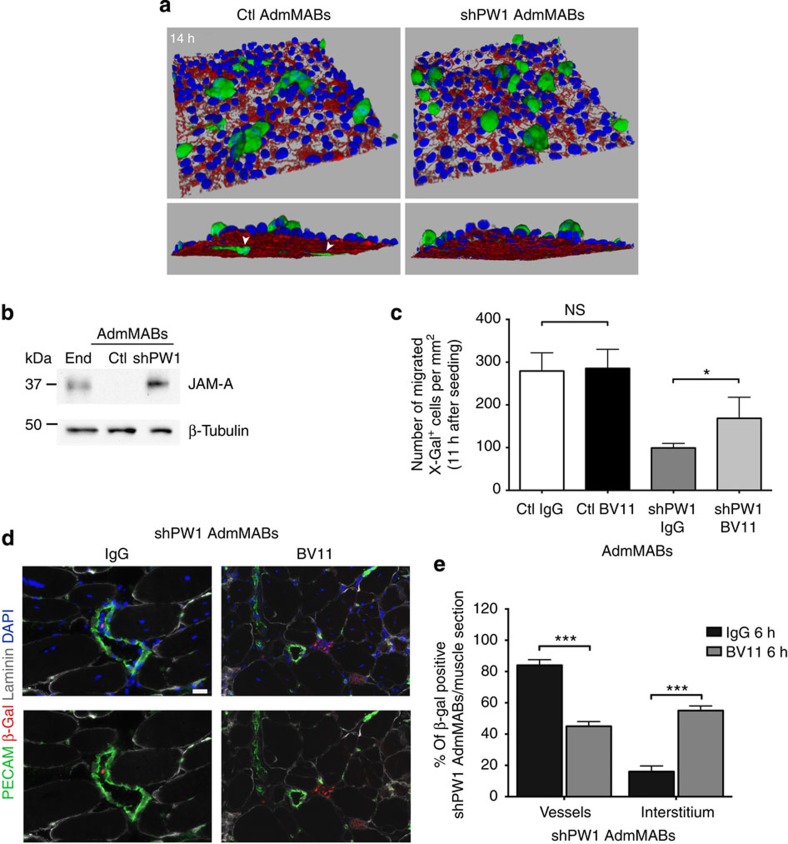
Impairment of JAM-A rescues the shPW1 AdmMABs transmigration *in vitro* and *in vivo*. (**a**) Time-course of 6-carboxyfluorescein diacetate (6-CFDA)-labelled Ctl and shPW1 AdmMABs transmigration across H5V endothelial cells seeded onto collagen matrix. Ctl and shPW1 AdmMABs were fixed at different time points during the transmigration assay and then the endothelial junctions were stained with anti-VE-cadherin (red) and nuclei (DAPI, blue). Three-dimensional reconstructions of a confocal *z*-stack taken after 14 h of Ctl and shPW1 AdmMABs transmigration are shown. The white arrowheads highlighted the portion of MABs under the endothelium. *xyz* field of view dimension 238.1 × 238.1 × 38.1 μm. (**b**) Western blot of JAM-A expression in Ctl and shPW1 AdmMABs. End, lung endothelial cells. β-Tubulin was used to normalize. (**c**) n-LacZ Ctl and shPW1 AdmMABs were pre-treated (2 h) with non-related IgG (20 μg ml^−1^) and JAM-A neutralizing antibody (anti-JAM-A mAb, 20 μg ml^−1^, BV11), respectively. Following this, cells were added to the upper chamber and allowed to migrate for 11 h. Migrated MABs on the lower side of the filters (X-Gal blue nuclei) were fixed and counted. Quantification of migrated MABs per area is shown. Data are means (±s.d.) from five independent experiments run in triplicate. *P<0.05, NS, not significant, unpaired one-way *t*-test. For figure in **d**, n-LacZ shPW1 AdmMABs were pre-treated (2 h) with non-related IgG (20 μg ml^−1^) and JAM-A neutralizing antibody (anti-JAM-A mAb, 20 μg ml^−1^, BV11), respectively. After antibody incubation, the n-LacZ shPW1 AdmMABs were intra-arterial transplanted in scid-*mdx* mouse. (**d**) Immunofluorescence staining for laminin (grey), β-gal (red), PECAM (green) and nuclei (DAPI, blue) on serial transverse sections of gastrocnemius muscle, 6 h after transplantation. Scale bar, 25 μm. The picture is representative of results obtained from two independent experiments (*n*=4 of mice used). The result from this analysis has been quantified in the graph (**e**). Values are plotted as % of β-Gal positive shPW1 AdmMABs per muscle section associated to vessel (PECAM positive), inside or outside (interstitium) the myofibres (by using laminin as reference). Statistical analysis has been performed comparing the BV11-treated shPW1 AdmMABs column with the respective IgG column for the different markers. ***P<0.0005.

To understand the basis of the impairment of migration and the dynamics of MAB transmigration through endothelial cells in the absence of PW1, we used live-cell imaging by confocal microscopy to follow this process at cellular level. Both Ctl and shPW1 AdmMABs were treated with a 6-carboxyfluorescin diacetate to allow MABs crossing the endothelial monolayers *in vitro*. We found that while shPW1 AdmMABs make contact with the endothelium, by 14 h, they are unable to cross the monolayer and retain a spherical shape. In contrast, Ctl AdmMABs are able to elongate pseudopods through the endothelial monolayer and start to transmigrate (Fig. 6a). To unravel the possible molecular mechanism at the basis of this phenomenon, we performed a microarray analysis (Affimetrix) comparing Ctl versus shPW1 AdmMABs. We focused on the most differentially expressed genes between shPW1 and Ctl AdmMABs. As shown in Supplementary Fig. 5a, we confirmed that cycE1 (CCNE1) and cycE2 (CCNE2) were upregulated in the absence of PW1. Siah1B, which has been described to bind PW1 in the p53-pathway^18^, is upregulated in the shPW1 AdmMABs. In addition, effectors underlying MAB homing represented by SDF1 (CXCL12) and its receptor (CXCR4)^26^ were deregulated in shPW1 AdmMABs. These results provide a mechanistic basis for MAB impairment to home towards damaged muscle. Intriguingly, in shPW1 AdmMABs, we observed a downregulation of muscular M-Cadherin (CDH15) and an upregulation of N-Cadherin (CDH2), normally expressed in neural and mesoderm cells, including undifferentiated myoblasts. Among the different adhesion molecules potentially involved in MAB extravasation, we focused upon the adhesion molecule JAM-A since it is upregulated in the absence of PW1 (Supplementary Fig. 5a). JAM-A is a tight junction molecule expressed primarily in endothelial and epithelial cells, leukocytes, dendritic cells and platelets. We found this result particularly intriguing as we recently demonstrated that JAM-A inhibition in endothelial cells reduces junction tightening, thus allowing MAB extravasation^27^. We therefore investigated whether PW1, through JAM-A repression, has a role in AdmMAB vessel transmigration. We confirmed that JAM-A was upregulated in shPW1 AdmMABs as compared with Ctl cells, both at messenger RNA (Supplementary Fig. 5b) and an even more pronounced difference at the protein level (Fig. 6b and Supplementary Fig. 5c). Notably, the non-competent AdmMAB clones (that is, the N−) showed the same regulation pattern of JAM-A (Supplementary Fig. 5d,e). We therefore performed a transmigration assay on Ctl and shPW1 AdmMABs pre-incubated with a blocking antibody for JAM-A (BV11) and an IgG as control. The interference with JAM-A in the shPW1 AdmMABs resulted in a 1.5-fold rescue in the capacity of shPW1 AdMABs to transmigrate (Fig. 6c). In addition, a comparable rescue of AdmMAB ability to transmigrate was achieved in non-competent N− AdmMAB clone following JAM-A blocking antibody (Supplementary Fig. 5f).

To assess if this rescue also occurs *in vivo*, shPW1 AdmMABs *nLacZ* were pre-incubated with the JAM-A blocking antibody (BV11) or an IgG control, followed by injection into the femoral artery of dystrophic immunodeficient scid-*mdx* mice, which were then sacrificed 6 h after injection. As shown in Fig. 6d,e, JAM-A function impairment in shPW1 AdmMABs led to a decrease in the percentage of shPW1 AdmMABs inside the vessels and to an increase of their presence in the interstitium, thus suggesting a rescue in their ability to transmigrate even *in vivo*, 6 h after transplantation.

Overall, these data highlight a fundamental role of PW1 for the other key feature of MABs: absence of PW1 impairs MAB ability to cross the vessel wall and reach the muscle through a mechanism that leads to the upregulation of JAM-A.

### PW1 regulates JAM-A by a direct binding to its promoter

**Figure 7:**
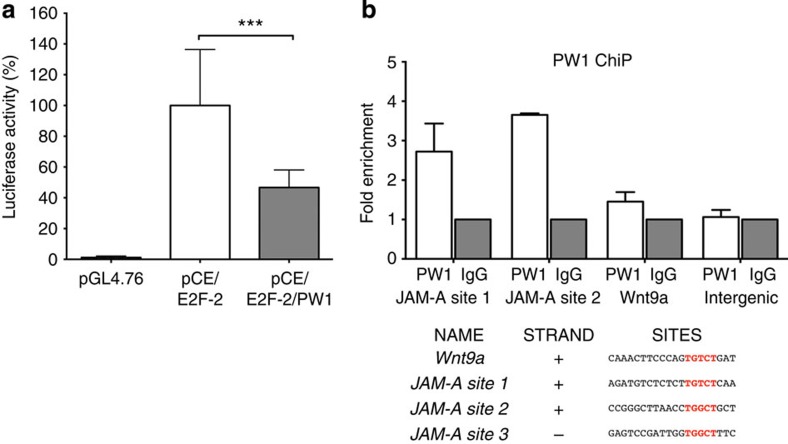
PW1 acts through a mechanism involving the direct binding to JAM-A promoter. (**a**) Luciferase activity was measured in triplicates 48 h after NIH3T3 transfection with pGL4.76 vector alone or together with pLuc-cycE/E2F-2 (pCE/E2F-2) or pLuc-cycE/E2F-2 plus PW1 (pCE/E2F-2/PW1). Data are means of 5 independent experiments, each of these was run in triplicate, and expressed as % of luciferase activity relative to the (pCE/E2F-2) signal. All the values have been normalized to the pGL4.76 signal. All data are represented as means ± s.d. from 5 independent experiments. ***P<0.0005, unpaired one-way *t*-test. (**b**) PW1 chromatin immunoprecipitation assay on growing AdmMABs to test two different putative PW1 binding sites on *JAM-A* promoter (JAM-A site 1 and site 2). Binding on *Wnt9a* promoter and on an intergenic region were checked as a predictive positive and negative controls, respectively. Data are means of two independent experiments and expressed as fold enrichment relative to the IgG signal.

PW1 has recently been shown to function as a transcription factor with a DNA-binding motif AGTnnCnnnTGGCT^20^. Our work suggests a mechanism in which PW1 regulates cycE and JAM-A. Using the Genomatix Matinspector software, we identified two different regions on the minus strand of the *cycE* promoter containing the core sequences necessary for PW1 binding. However, due to a >80% GC content, we were unable to obtain reproducible data confirming that PW1 directly binds to these sites. Nevertheless, we measured the luciferase activity in NIH3T3 cells co-transfected with pLuciferase-*cycE* −94/+263 promoter^28^ (pCE), pE2F-2 (a well-known cycE-activator) and pGL4.76 vector (as control of transfection efficiency). A significant reduction of luciferase activity was observed when NIH3T3 cells were co-transfected with PW1, thus suggesting a repressive role of PW1 on *cycE* promoter (Fig. 7a). For JAM-A, we identified two hypothetical binding regions in the *JAM-A* promoter (JAM-A site 1 and 2) (Fig. 7b). Chromatin immunoprecipitation (ChiP) assays performed on these PW1 binding domains on *JAM-A* promoter confirmed a direct binding of PW1 to *JAM-A* (Fig. 7b) providing a mechanistic basis for PW1-mediated regulation of JAM-A seen in our studies.

### PW1 levels correlate with human MAB competence

**Figure 8:**
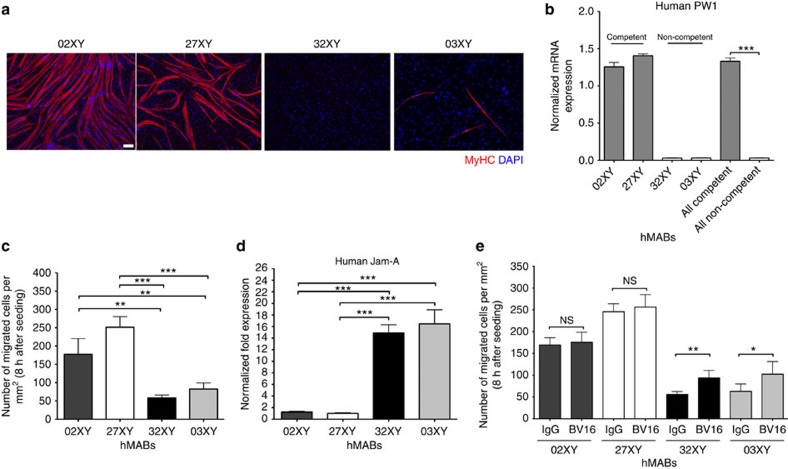
PW1 levels strongly correlate with the myogenic and transmigration ability of human MABs. (**a**) Immunofluorescence analysis for MyHC (red) in four different populations of hMABs. DAPI was used to stain the nuclei. Scale bar, 100 μm. (**b**) Human PW1 expression by qRT–PCR on four different populations of hMABs divided in competent (02XY and 27XY) and non-competent (32XY and 03XY) on the basis of the myogenic property shown in **a**. Values are plotted as relative expression and normalized to GAPDH expression. Each assay was performed in triplicate. All data are represented as means ± s.d. ****P<0.0001, unpaired one-way *t*-test. (**c**) HUVECs endothelial cells were seeded on gelatin-coated filters. Four different polyclonal hMABs, previously labelled with 3.33 μM 6-carboxyfluorescein diacetate (6-CFDA), were added to the upper chamber and allowed to migrate for 8 h. Migrated hMABs on the lower side of the filters (fluorescein isothiocyanate-positive cells) were fixed and counted. Quantification of migrated hMABs per area is shown. Data are means (±s.d.) from five independent experiments, each of these was run in triplicate. **P<0.005, ***P<0.0005, unpaired one-way *t*-test. (**d**) Human JAM-A expression by qRT–PCR on hMABs. Values are plotted as relative fold expression and normalized to GAPDH expression. All data are represented as means ± s.d. Each assay was performed in triplicate. ***P<0.0005; unpaired one-way *t*-test. (**e**) The HUVEC endothelial cells were seeded on gelatin-coated filters. Competent (02XY and 27XY) and non-competent (32XY and 03XY) hMABs, previously pre-incubated with the anti-human JAM-A BV16 (12 μg ml^−1^) and the IgG (12 μg ml^−1^) as control, were then labelled with 3.33 μM 6-CFDA. Cells were then added to the upper chamber and allowed to migrate for 8 h. Migrated hMABs on the lower side of the filters were fixed and counted. Quantification of migrated hMABs per area is shown. Data are presented as means (±s.d.) from five independent experiments, each of these was run in triplicate. **P<0.005, *P<0.5, unpaired one-way *t*-test.

In the perspective of a potential future clinical use of this marker, we explored whether PW1 may represent a marker of myogenic potency also in human MABs (hMABs). In such a case it would be possible to test PW1 expression to identify the best hMAB donors in terms of myogenic competence and ability to cross blood vessels. Therefore, we surveyed PW1 expression levels in different hMAB cell lines. Twelve polyclonal populations had been previously isolated^29^ and tested for their ability to differentiate in culture. Four representative populations (Fig. 8a) were selected and tested for PW1 expression levels by qRT–PCR. As shown in Fig. 8b, PW1 was strongly expressed only in myogenic competent populations of hMABs. In addition, transmigration assays on human umbilical vein endothelial cells (HUVECs) performed on both competent (02XY and 27XY) and non-competent (32XY and 03XY) differentiating hMABs confirmed that the ability to cross the vessel wall strongly correlated with levels of human JAM-A (Fig. 8c,d). We also performed the transmigration assay on competent (02XY and 27XY) versus non-competent (32XY and 03XY) hMABs pre-incubated with a blocking antibody specific for the human JAM-A (BV16) and an IgG as control. The interference with human JAM-A in the non-competent hMABs resulted in almost twofold rescue in their capacity to transmigrate (Fig. 8e). Taken together, these data confirm the utility of PW1 as a screening molecule/biomarker in future cell therapy protocols on the basis of MAB transplantation for muscle disorders.

## Discussion

In the last decade, MABs have been isolated from different species (mouse, dog and human) at different developmental stages (from mouse embryo to adult humans, including also iPS cells)^1^,^4^,^6^,^9^,^30^. Extensive analyses have shown that MABs can differentiate into skeletal muscle and cross the vessel wall to target the damaged muscle following systemic injection^2^,^4^,^5^,^6^,^9^. These key properties have provided the foundation for testing MABs as a medicinal product for stem cell-mediated therapies. Even though these cells are being tested in clinic, it has been a challenge to determine, without lengthy studies, both whether different preparations of MABs share a high level of competence and what may account for the variability of competence between individual preparations. Microarray analyses performed on different MABs revealed that PW1 is expressed in all MABs isolated so far. The utility of PW1 as a marker of MAB competence proposed here is consistent with the recent observation that PW1 can be used to identify multiple adult stem and progenitor cell populations in different tissues^16^.

Silencing of PW1 in a polyclonal population of adult mouse MABs interfered with the two main features of these cells: shPW1 AdmMABs showed not to be able to differentiate in skeletal muscle and to cross the vessel wall. These results were also verified in different clones of embryonic and adult mouse MABs. Previous work from our laboratory showed that the paired box/homeodomain transcription factor Pax3 is necessary for embryonic mesoangioblast differentiation^21^. Surprisingly, silencing of Pax3 in the polyclonal population of AdmMABs did not lead to any phenotype or change in terms of muscle differentiation. Although our data revealed that Pax3 acts upstream of PW1 in regulating embryonic MAB skeletal differentiation (Supplementary Fig. 2c), this is not the case in adult mMABs. We propose that Pax3 is only required for embryonic myogenic competence whereas PW1 is required at all developmental stages. As the absence of PW1 interferes with MAB function, we also investigated the possible mechanisms through which PW1 may act. PW1 is a multidomain protein that contains 12 Cys_2_His_2_ Kruppel-type zinc-finger motifs as well as two proline-rich periodic repeats which predict the ability of binding to DNA^11^. We demonstrated previously that PW1 regulates two key cell stress pathways via interaction with the TNF receptor-associated factor2 (TRAF2) and p53-mediated cell death through direct interaction with Siah1 (Seven in absentia homolog1) and Bcl2-associated X (BAX) proteins^17^,^18^. PW1 was also demonstrated to inhibit Wnt signalling by promoting β-catenin degradation^19^. Here, we demonstrate that the absence of PW1 causes a block in mesoangioblast myogenic differentiation through a sustained cycE expression that, in turns, drives MyoD to degradation via the proteasome machinery. Nevertheless, the levels of MyoD in proliferating Ctl AdmMABs, which express high levels of cycE, suggests that cycE acts in concert with other regulatory pathways in mediating the level of MyoD in shPW1 AdmMABs. We note, however, that inhibition of the CyclinE-cdk2 activity through Roscovitin treatment is sufficient to rescue MyoD protein in growing shPW1 cells, thus demonstrating that this pathway has a major role in MABs.

Mesoangioblasts are able to cross the vessel wall, which provides an advantage over satellite cells as a donor stem cell for systemic cell therapy in treating muscular dystrophies. We observed that PW1 silencing leads to a major adhesion to the endothelium preventing AdmMABs extravasation. The microarray analysis comparing Ctl and shPW1 cells revealed marked changes in the levels of gene expression underlying cell adhesion and cellular homing^26^. We focused on JAM-A in this study as endothelial JAM-A inhibition increases MAB transmigration upon systemic delivery^27^. Surprisingly, we observed a robust increase of JAM-A in the absence of PW1 and a corresponding loss of trans-vessel migration capacity (Fig. 5e) that can be rescued following JAM-A interference (Fig. 6d) in the shPW1 AdmMABs. Taken together, these data suggest that PW1 acts upstream in the JAM-A pathway, thus influencing MAB competence to cross blood vessel wall.

A recent report demonstrated that PW1 can act as a transcription factor with the DNA-consensus motif (AGTnnCnnnTGGCT) where PW1 is able to bind and repress specific target genes in embryonic brain^20^. Our analyses of the *cycE* promoter identified two different regions present on the minus strand of the *cycE* promoter, containing the core sequence necessary for PW1 binding. Although we were unable to demonstrate a direct binding, we observed a significant reduction of *cycE* promoter activity following PW1 expression (Fig. 5f). In addition, we identified two PW1 binding regions in *JAM-A* promoter (JAM-A site 1 and 2) that are directly bound by PW1, thus providing a mechanistic basis for PW1-mediated regulation of JAM-A seen in our studies. While PW1 has been shown to exert numerous non-transcriptionally mediated events, our results provide a mechanistic framework in which PW1 acts at least in part as transcription factor to confer key properties to MABs.

Among the multiple obstacles in using allogeneic cell transplantation for therapeutic ends, a key challenge is to identify the most appropriate donor cell. In addition to requirements related to the immune system such as HLA compatibility^31^, the optimal donor cell must cross the vessel wall with high efficiency and once resident in the damaged muscle tissue, it must be able to participate in the myogenic programme. At present, screening the competence of each preparation of MABs is time consuming and costly requiring both *in vitro* and ultimately *in vivo* assays. The striking correlation between the level of PW1 expression in MABs and their ability to differentiate into muscle and to cross the endothelium in numerous cell preparations across species and age suggests that assessing PW1 levels can substitute as rapid and single step assay for screening donor cell preparations for therapeutic value. Since PW1 expression identifies multiple adult stem/progenitor cell populations, we also propose that PW1 is not only a marker, but also regulates competence in other stem cells, such as neural and skin stem cells and can be used as a pre-clinical screening tool in a larger stem cell therapy-based context. Future studies will shed light on this possibility, thus broadening and reinforcing the significance of this study.

## Methods

### Cell cultures and drug treatments

We used well-established mouse adult mesoangioblasts (AdmMABs) and D16 embryonic mMAB cell line^1^,^4^,^29^. These cells were maintained in Dulbecco’s Modified Eagle’s Medium (DMEM, Sigma) with 20% fetal bovine serum (FBS, Lonza), 2 mM L-glutamine, 100 IU ml^−1^ penicillin and 100 mg ml^−1^ streptomycin (G+PS). Muscle differentiation was induced, after 24 h in growing medium (GM), by incubating cells in DMEM with 2% horse serum (Lonza) and G+PS. Cell cultures were incubated at 37 °C and 5% CO_2_. For D16 muscle differentiation, D16 cells were co-cultured with the L6 rat myoblasts at a 1:10 ratio. 37 clones were isolated from the polyclonal population of AdmMABs by limiting dilution. Cells have been diluted and one-cell-per-96-wells plated. Clones were selected on the basis of their differentiation capacity and among these, six most representative ones have been chosen: non-competent clones (with a limited differentiation ability) were named L, N, O; competent clones (with a remarkable differentiation ability) were named C, D, G.

Human mesoangioblasts (hMABs) were isolated from muscle biopsies under the protocol ‘Evaluation of regenerative properties of human mesoangioblasts’, submitted to the San Raffele Ethical Committee in 2007. Once isolated human cells have been grown in Megacell DMEM (Sigma), 5% FBS, 2 mM glutamine (Sigma), 0.1 mM β-mercaptoethanol (Gibco), 1% non-essential amino acids (Sigma), 5 ng ml^−1^ human bFGF (Sigma),100 IU ml^−1^ penicillin and 100 mg ml^−1^ streptomycin (G+PS). Skeletal muscle differentiation was induced seeding cells on Matrigel-coated dishes and, after 24 h in growing medium, by incubating cells in DMEM supplemented with 2% horse serum^29^. Adult dog skeletal muscle MABs were isolated from muscle biopsies of golden retriever dogs and were maintained in culture in DMEM (Sigma) with 20% FBS and G+PS. Mouse embryonic fibroblasts NIH3T3 (ATCC) and mouse H5V endothelial cells (ATCC) were grown in DMEM with 10% FBS and G+PS. HEK293T (ATCC) were cultured in Iscove Modified Eagle’s Medium (Sigma) with 10% FBS (Lonza) and G+PS. HUVECs were grown in MCDB-131 (Gibco) with 20% FBS, 10 μg ml^−1^ heparin (Sigma-Aldrich), 5 μg ml^−1^ ECGS (endothelial cell growth factor human, Sigma-Aldrich) and G+PS.

Proteasome inhibitor MG132 (Sigma-Aldrich) was used at 50 μM for 3 and 5 h; Roscovitine (Calbiochem) was used at 5 μM for 5 h.

To identify proliferating cells, we performed incorporation assay with 5-bromo-deoxyuridine (BrdU; GE Healthcare Life Sciences). MABs were incubated with 50 μM BrdU for 1 h before fixation.

### Mice and MAB transplantation

All animals studied here were housed in San Raffaele Scientific Institute animal house. Mice were kept in pathogen-free conditions and all procedures were conformed to Italian law and approved by the San Raffaele Animal Care and Use Committee. Intra-arterial cell transplantations have been performed in 5-month-old scid*-mdx* female mice, previously anaesthetized with Avertin (Sigma). A total of 10^6^ mMABs were diluted in 50 μl of phosphate-buffered saline (PBS) and injected into the isolated femoral artery. The animals were sacrificed 6 h or 1 month after the injection, then muscles were collected and analysed. For the *in vivo* rescue experiment, control and shPW1 cells were pre-incubated with the BV11 blocking peptide and control IgG (at 20 μg ml^−1^) for 2 h before transplantation.

### Lentiviral and retroviral vector production and cell transduction

The shPW1 was generated by Vectalys, Prologue Bioteck, France. Briefly, a forward primer (5′- CGCGACTGTACGAATGCAAAGATTTCAAGAGAATCTTTGCATTCGTACAGTTTTTTTCA -3′) was designed including the *PW1* core sequence (5′- ACTGTACGAATGCAAAGAT -3′) as well as MluI and NsiI sites for cloning. The reverse primer was designed as following 5′- AAAAAACTGTACGAATGCAAAGATTCTCTTGAAATCTTTGCATTCGTACAGT -3′. The shPW1 was generated by hybridization of oligonucleotides and inserted by MluI/NsiI cohesive ligation into a pLV-H1 lentiviral plasmid containing a RNA polymerase III promoter. The pLKO.1shPax3 and pLKO.1shCONTROL were purchased by Darmacon (Open Biosystems). The lentiviral particles were produced by transfecting the HEK 293 cells with 32 μg of the lentiviral vectors together with the packaging plasmids pMDL (12,5 μg), pRev (6.25 μg) and pVSV-G (9 μg). After 36 h from transfection, the supernatant was collected and concentrated following centrifugation at 20,000 r.p.m. for 2 h. For viral transductions, 10^5^ MABs were seeded in 35-mm dishes and infected at a multiplicity of infection of 100 with the lenti shPW1 in polybrene presence (8 μg ml^−1^). Infection was allowed to proceed overnight. The day after, medium was changed and the selective agent, puromicin (Sigma), was added to the cultures.

The retroviral vectors used, pBabe Hygro, pBabeHygroMyoD and pBabeHygroMyoDSP3, are published in ref. 25. For retroviral transductions, 10^5^ shPW1 AdmMABs were seeded in 35-mm dishes and 1 ml of retroviral stock was added; infection was allowed to proceed overnight. After overnight incubation, the medium was changed and the selective agent, hygromycin B (Sigma), was added to the cultures. The MABs were transduced with third generation lentiviral vectors expressing nuclear LacZ at the multiplicity of infection of 100, after previous incubation with Polybrene (8 μg ml^−1^).

### Transfection

The MABs were transfected with the Lipofectamine LTX transfection reagent (Invitrogen). The NIH3T3 cells were transfected using Lipofectamine 2000 (Invitrogen). The plasmids used were the pEGFP and pEGFPW1 already published in ref. 11.

### Immunofluorescence and histology

Cell cultures were washed twice with PBS and fixed with 4% paraformaldehyde at 4 °C for 10 min. Muscle samples were frozen in liquid nitrogen-cooled isopentane (VWR, Italy) and serial 7-μm-thick sections were cut with a Leica cryostat (Leica Microsystems) and fixed with 4% paraformaldehyde at 4 °C for 10 min.

Cells and tissue were permeabilized with 0.2% Triton X-100 (Sigma), 1% bovine serum albumin (BSA, Sigma) in PBS for 30 min at room temperature (RT), then blocked with 10% goat serum for 30 min at RT. Samples were incubated overnight at 4 °C with the following primary antibodies: mouse anti-MyHC 1:2 (MyHC; MF20, Developmental Studies Hybridoma Bank, USA), rat anti-PECAM 1:2 (CD31- Developmental Studies Hybridoma Bank, USA), rabbit anti-β-Gal 1:500 (Cappel), mouse anti-MyoD 1:100 (Dako), rabbit anti-laminin 1:300 (Sigma), mouse anti-dystrophin 1:50 (Monosan), goat anti-mouse VE-Cad 1:400 (Santa Cruz). After incubation, the samples were washed with 0.2% Triton X-100, 1% BSA in PBS and then incubated with the secondary antibodies 1:500 (Jackson Laboratories) and 4',6-diamidino-2-phenylindole (DAPI) 1:500 (Sigma) for 45 min at RT in PBS. After two washes, dishes or slides were mounted using fluorescent mounting medium (Dako) and watched under fluorescence microscopes (Leica-DMI6000B). For the immunostaining for PW1, the cells were fixed with 4% paraformaldehyde at 4 °C for 10 min. After three washes, the cells were permeabilized with methanol at −20 °C for 6 min and blocked with 5% goat serum and 2% of BSA (Sigma) in PBS for 3 h at RT. Samples were incubated overnight at 4 °C with PW1 antibody (1:3,000) in 5% goat serum and 2% of BSA (Sigma) in PBS. After incubation, the samples were washed with 0.1% BSA (Sigma) in PBS for 15 min at RT and 5% BSA (Sigma) in PBS for 15 min at RT. Then the cells were incubated with the secondary antibody 1:500 (Jackson Laboratories) and DAPI 1:500 (Sigma) for 2 h at RT in 5% goat serum and 2% of BSA (Sigma) in PBS. After incubation with the secondary antibody, the samples were washed with 0.1% BSA (Sigma) in PBS for 15 min at RT and 5% BSA (Sigma) in PBS for 15 min at RT, mounted using fluorescent mounting medium (Dako) and watched under fluorescence microscopes (Leica-DMI6000B).

Tissue sections were stained with eosin (Sigma) and X-gal (Invitrogen) according to standard protocols.

### Microarray

The Microarray and the following analysis were performed by the DNA-Affimetrix facility at IFOM Campus. RNA from Ctl and shPW1 AdmMABs was isolated using commercial homogenization (QIAshredder) and purification (RNeasy Mini Kit) reagents (Qiagen). Quality control (QC) of the RNA samples was performed using an Agilent Bioanalyzer 2100 (Agilent Technologies). Two different RNA extractions were processed for each of the cell lines under analysis, and each sample was labelled and hybridized to a Mouse Gene 1.0 ST Genechip array according to the manufacturer’s specifications (Affymetrix). Data were analysed using Partek Genomics Suite v6.3 software (RMA algorithm). Differentially expressed genes were identified through analysis of variance, using a fold change cut-off >1.2 and a *P* value of 0.05. Microarray data have been deposited in Gene Expression Omnibus (GEO) under accession code GSE64334.

### Quantitative PCR with reverse transcription

Total RNA was isolated using NucleoSpin RNA XS Kit (Macheray-Nagel) and was retro-transcribed to cDNA with the iScript Reverse Transcription Supermix (Bio-Rad), according to the manufacturer’s instructions. Each cDNA sample was amplified in triplicate using the iTaq Universal SYBR Green Supermix (Bio-Rad) or the SSo Advanced SYBR Green Supermix (Bio-Rad).

The primers used are detailed in the Supplementary Table 1.

Concerning human JAM-A qRT–PCR, 400 ng of RNA was reverse transcribed with random hexamers (High Capacity cDNA Archive Kit; Applied Biosystems) in accordance with the manufacturer’s instructions. cDNA (5 ng) was amplified in triplicate in a reaction volume of 15 μl with the TaqMan Gene Expression Assay (Applied Biosystems) and an ABI/Prism 7900 HT thermocycler. For any sample the expression level, normalized to the housekeeping gene encoding *GAPDH*, was determined with the comparative threshold cycle (Ct) method.

### Western blot

Proteins were extracted with a solution composed of 1% NP40 (Sigma), 0.5% sodium deoxycholate (Sigma), 1 mM PMSF (phenylmethylsulfonyl fluoride), 5 mM sodium orthovanadate (Sigma), proteases and phosphatases inhibitors. The western blot for PW1 was performed as follows. Equal amounts of proteins were run at 100 V and then transferred on nitrocellulose membranes with the Iblot Dry Blotting System (Life Technologies) for 10 min. The membrane was blocked with 5% skimmed milk, 0.05% Tween-20 (Sigma) in PBS for 1 h while shaking at RT and incubated with primary antibody for PW1 (1:10,000) in 5% skimmed milk, 0.05% Tween-20 (Sigma) in PBS overnight in agitation at 4 °C. After the primary antibody incubation, the membrane was washed four times for 10 min each with 0.05% Tween-20 (Sigma) in PBS (T-PBS) at RT in agitation. Then it was incubated with secondary antibody conjugated to peroxidase 1:10,000 (Bio-Rad) in 5% skimmed milk in T-PBS for 45 min at RT in agitation. After four washes for 10 min each with T-PBS at RT in agitation, the membrane was incubated in ECL chemiluminiscence reagents following the manufacturer’s instructions (Amersham). For all the other markers, the western blot was performed as follows. Equal amounts of proteins were run at 100 V and once the proteins were separated, the gel was transferred on nitrocellulose membranes in according with each molecular weight. The membrane was blocked with 5% skimmed milk in T-PBS for 1 h in agitation at RT and incubated with primary antibody in 5% skimmed milk in T-PBS overnight in agitation at 4 °C. The antibodies used were: mouse anti-MyHC 1:2 for cultured cells, 1:10,000 for tissues (MF20-Hybridoma Bank), mouse anti-MyoD 1:1,000 (Dako), rabbit anti-Myf5 1:500 (Tebu-Bio), mouse anti-Myogenin 1:3 (IF5D- Hybridoma Bank), mouse anti-dystrophin 1:500 (Monosan), mouse anti-Pax3 1:2 (Hybridoma Bank), mouse anti-Pax7 1:3 (Hybridoma Bank), mouse anti-cycE 1:1,000 (Santa Cruz), rat anti JAM-A^32^ 1:250, mouse anti β-tubulin 1:5,000 (Covance), mouse anti-GAPDH 1:5,000 (Biogenesis), mouse-Vinculin 1:2,500 (Sigma-Aldrich). After the primary incubation, the membrane was washed four times for 10 min each with T-PBS at RT in agitation. Then it was incubated with secondary antibody conjugated to peroxidase 1:10,000 (Bio-Rad) in 5% skimmed milk in T-PBS for 45 min at RT in agitation. After four washes for 10 min each with T-PBS at RT in agitation, the membrane was incubated in ECL chemiluminiscence reagents following the manufacturer’s instructions (Amersham).

All the uncropped scan of western blots are shown in Supplementary Information.

### ChiP assay

For the ChiP assay, 3 × 10^6^ AdmMABs were maintained in growth condition for 2 days. Protein-DNA was crosslinked with a solution of 1% formaldehyde (Sigma-Aldrich) in DMEM for 10 min. Crosslink was blocked with 0.125M glycine (Sigma-Aldrich) in PBS, for 10 min while shaking. After two washes with PBS, cells were scraped with PBS containing protease inhibitors and PMSF. The suspension was centrifuged for 15 min at 1,500 *g*, 4 °C, the supernatant removed and the cell pellet was dounced 20 times in a 7 ml pestle dounce in swelling buffer. Swollen cells were then centrifuged for 10 min at 5,000 *g*, 4 °C and the nuclei pellets were sonicated on ice-cooled water with a Bioruptor Sonicator (Diagenode) for a total of 15 min, with repeated cycles of 15 s of sonication time and 15 s of resting. Samples were finally centrifuged for 10 min at 12,000 *g*, 4 °C to eliminate cell debris. Chromatin-enriched supernatant was precleared for 2 h with protein G Sepharose (PrG, 15 μl per sample) (Amersham) and rabbit serum (2,5 μl per sample), then for 2 h with PrG previously blocked with BSA (10 μg ml^−1^) and salmon sperm (1 μg ml^−1^, Sigma-Aldrich), on a rotating platform at 4 °C. Chromatin was than incubated overnight with the following primary antibodies (3 μg for each immunoprecipitation): rabbit anti-PW1^33^, normal rabbit IgG (SantaCruz). The day after, immunocomplexes were precipitated by addition of blocked PrG, for 3 h on rotating platform at 4 °C. After immunoprecipitation, samples were centrifuged for 2 min at 12,000 *g*, 4 °C. Samples were then repeatedly washed to eliminate aspecific bindings. The antibody–protein-DNA complexes were eluted twice for 10 min at 65 °C. Eluted samples were incubated overnight at 65 °C with 10 μg RNase (Sigma-Aldrich) and 200 mM NaCl (Sigma-Aldrich), to reverse formaldehyde crosslinks. Finally, DNA was purified from proteins with a Proteinase K (Sigma-Aldrich) treatment (20 μg per sample) for 3 h at 50 °C, and then DNA extracted through phenol–chloroform. Immunoprecipitated DNA was subjected to qRT–PCR. The primers used are detailed in the Supplementary Table 2.

### Luciferase activity detection

The pLuciferase-cycE −94/+263 (pCE), published in ref. 28, and pCMVHAE2F-2 (E2F-2) were kindly provided by G. Piaggio (IRE, Rome) and M. Crescenzi (ISS, Rome), respectively. pGL4.76 was purchased by Promega. NIH3T3 cells were co-transfected with pCE, E2F-2, pGL4.72 with or without PW1 according to the Lipofectamine 2000 manufacturer’s protocol (Invitrogen). The pGL4.76 vector was used as an internal control for transfection efficiency. Luciferase activity was detected according to the Dual-Luciferase Reporter Assay System (Promega).

### *In vitro* transmigration assay

The 8 μm transwell filters (Corning) were previously coated with glutaraldehyde-crosslinked gelatin to enhance endothelial cell adhesion: the culture supports were incubated for 1 h at RT with 0.5% (for murine endothelial cells) or 1.5% (for HUVECs) gelatin (Sigma-Aldrich), followed by crosslinking with 2% glutaraldehyde solution (Sigma-Aldrich) for 15 min at RT. The glutaraldehyde was then replaced by 70% ethanol. After 1 h, five washes with sterile PBS were followed by an overnight incubation with PBS containing 2 mM glycine. Before cell seeding, the slides were washed five times with sterile PBS. Endothelial cells H5V were plated to confluence in insert for 24 h. Ctl, shPW1, competent and non-competent AdmMABs, previously transduced with a Lentiviral vector expressing the nuclear LacZ, were plated in DMEM supplemented with 2% horse serum (Lonza) on the upper side of transwell chamber once the monolayer of H5V cells was complete (confluence of the endothelial monolayer was assessed by crystal-violet staining). After 11 h, migrated MABs on the lower side of the filter were fixed in 4% paraformaldehyde, stained with X-gal and counted using a phase microscope (five random fields of the lower face of the transwell membrane at × 10 magnification).

HUVECs were plated to confluence in insert for 72 h. Human MABs were labelled with 3.33 μM 6-carboxyfluorescein diacetate (Molecular Probes, Invitrogen) for 20 min at 37 °C. The fluorescent human MABs were added to the upper chamber in DMEM supplemented with 2% horse serum (Lonza). After 8 h, migrated MABs on the lower side of the filter were fixed in 4% paraformaldehyde and counted using a fluorescence microscope (five random fields of the lower face of the transwell membrane at × 10 magnification).

For the *in vitro* transmigration assay in the presence of monoclonal anti-mouse (clone BV11 (ref. 27)) or human JAM-A (clone BV16 (ref. 27)) MABs, both mouse or human MABs were pre-treated with the monoclonal anti-JAM-A antibody (BV11 at 20 μg ml^−1^ and BV16 at 12 μg ml^−1^) and human JAM-A in DMEM supplemented with 0,5% FBS for 2 h at 37 °C. After incubation, the cells were plated on the upper side of a transwell chamber and the lower side was supplemented with DMEM 0.5% FBS with the anti-JAM-A antibody. After 11 (for mMABs) or 8 (for hMABs) hours, migrated MABs on the lower side of the filter were fixed in 4% paraformaldehyde and counted using a microscope (five random fields of the lower face of the transwell membrane at × 10 magnification). The results show the number of migrated cells per mm^2^.

## Author contributions

C.B. performed the experiments with the assistance of S.A.; G.R. performed the ChiP assays; F.S.T. performed and analysed *in vivo* transplantation experiments; M.G. performed part of the experiments on JAM-A; R.T. isolated the different populations of hMABs; S.B. performed the differentiation assays on hMABs; G.Me. conceived and designed the experiments; G.Me. wrote the paper with assistance from G.C, D.S, G.Ma. and E.D.

## Additional information

**Accession codes**: Microarray data have been deposited in Gene Expression Omnibus (GEO) under accession code GSE64334.

**How to cite this article:** Bonfanti, C. *et al*. PW1/Peg3 expression regulates key properties that determine mesoangioblast stem cell competence. *Nat. Commun.* 6:6364 doi: 10.1038/ncomms7364 (2015).

## Supplementary Material

Supplementary Information Supplementary Figures 1-11 and Supplementary Tables 1-2
