# Supplementary material for: PW1/Peg3 expression regulates key properties that determine mesoangioblast stem cell competence
Source: Nat Commun. 2015 Mar 9;6:6364. doi: 10.1038/ncomms7364 (PMC4366533; doi:10.1038/ncomms7364)
Supplement: Supplementary Information — Supplementary Figures 1-11 and Supplementary Tables 1-2 [file ncomms7364-s1.pdf]

Supplementary Figure Legends

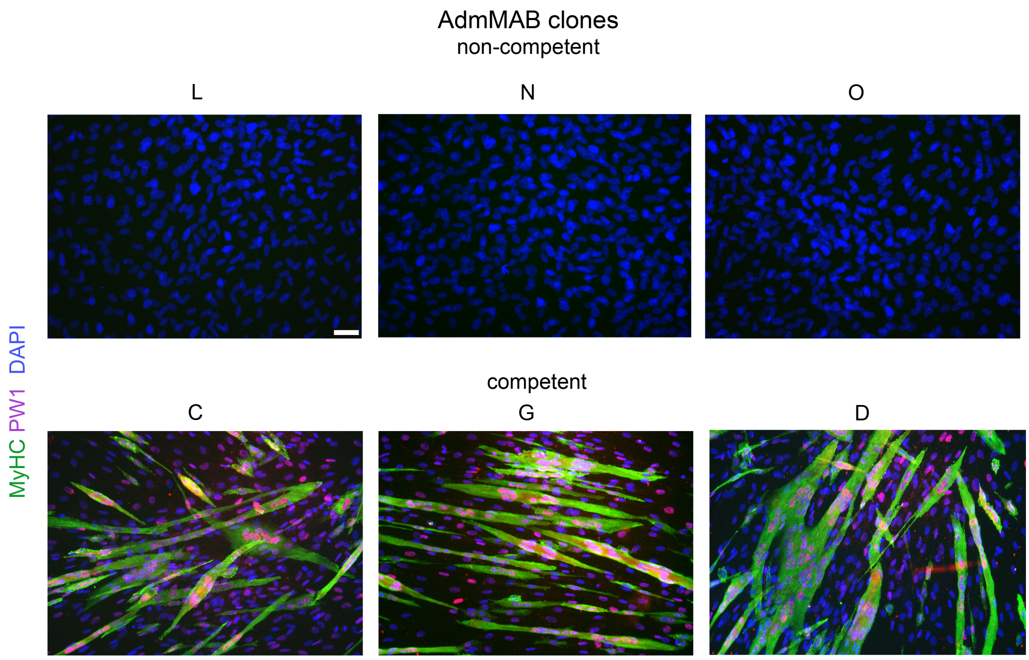

Supplementary Figure 1 Myogenic non-competent AdmMAB clones do not express PW1

Immunofluorescence staining for MyHC (green), PW1 (red) and nuclei (DAPI) has been performed on six clones of AdmMABs divided in competent (C, G, D) and non-competent (L, N, O) on the basis of their myogenic property. Scale bar 50μm.

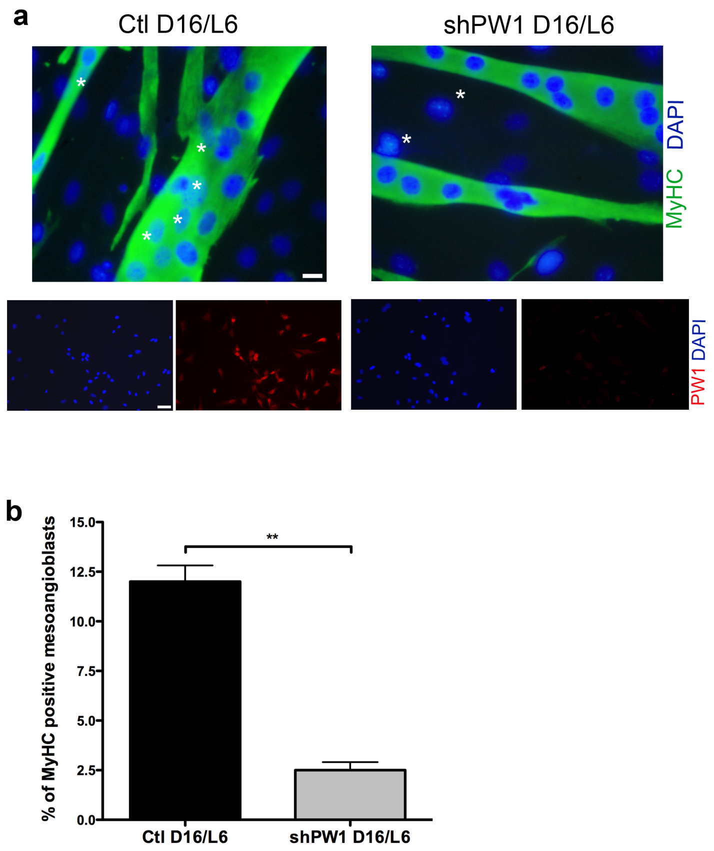

Supplementary Figure 2 Absence of PW1 in embryonic mMABs leads to muscle differentiation impairment

(**a**) Immunofluorescence staining for MyHC (green), PW1 (red) and nuclei (DAPI) has been performed on the embryonic clone of mMABs, D16, previously transduced with lentiviral scramble or shPax3 vectors. Ctl or shPax3 D16 mMABs have been following co-cultured with the rat L6 myoblasts and induced to differentiate in skeletal muscle. Mouse nuclei (asterisks) are distinguishable from rat nuclei for the typical DAPI staining, which highlights the nuclear dots. Scale bar 100 and 50 μm. The result from this analysis has been quantified in the graph (**b**). Values are plotted as % of MyHC positive mMABs out of total MyHC positive cells nuclei (rat-L6 cells- and mouse- D16).

Each assay was performed in triplicate. Data are represented as means +/- S.D. **$P < 0.005$, unpaired *t* Test.

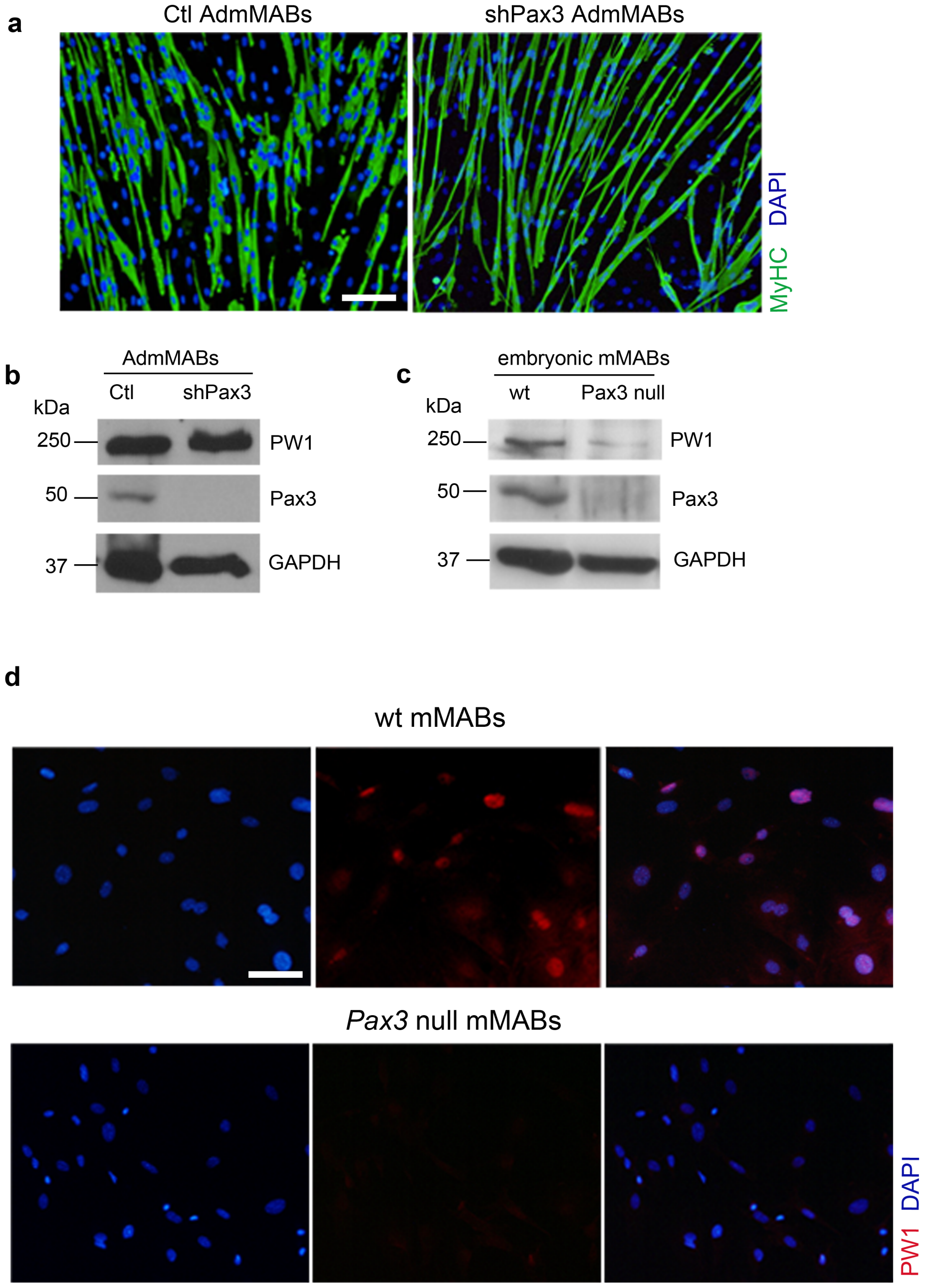

Supplementary Figure 3. Silencing of Pax3 in AdmMABs does not alter MAB myogenic competence

(**a**) Immunofluorescence staining for MyHC (green) and nuclei (DAPI) has been performed on AdmMABs, previously transduced with lentiviral scramble or shPax3 vectors. Scale bar 100 μm. (**b**) Western Blot analysis of Pax3 and PW1 in Ctl and shPax3 AdmMABs. GAPDH was used to normalize the amount of loaded proteins. (**c**)

Western Blot analysis of Pax3 and PW1 in wt and *Pax3* null polyclonal population of embryonic mMABs. GAPDH was used to normalize the amount of loaded proteins. (**d**) Immunofluorescence staining for PW1 (red) and nuclei (DAPI) has been performed on embryonic wt and *Pax3*null mMABs in growing condition. Scale bar 100 μm.

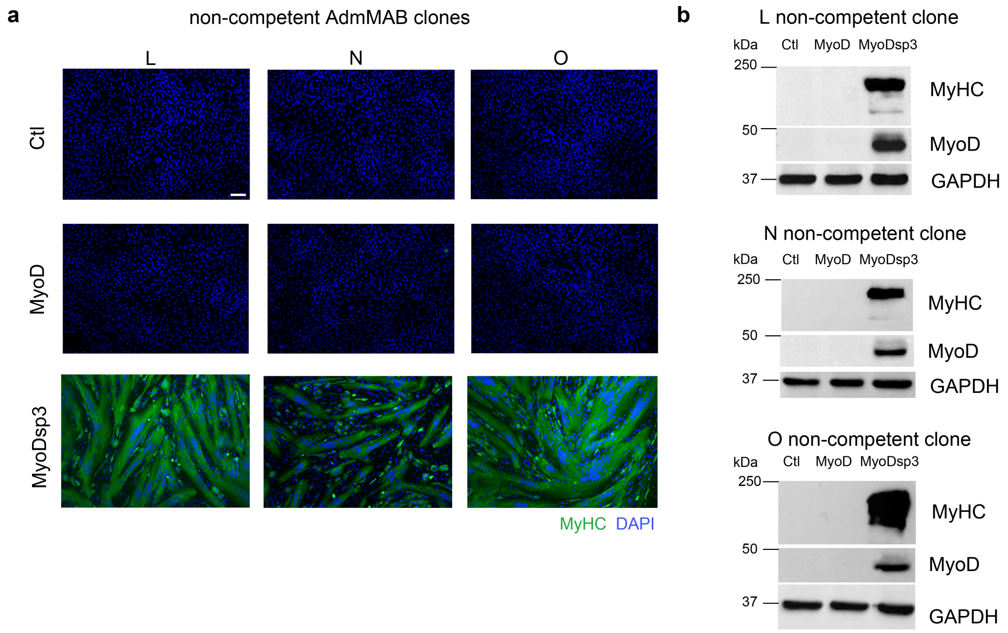

Supplementary Figure 4 Rescue of myogenic competence in non-competent AdmMABs clones by MyoDsp3

(**a**) Immunofluorescence staining for myosin heavy chain (MyHC, green), and nuclei (DAPI, blue) on non-competent AdmMAB clones (L, N, O) transduced with retroviral vector expressing *wt* MyoD (MyoD), mutated MyoD (MyoDsp3) and empty control vector (Ctl). Scale bar represents 100 μm. (**b**) Western Blot analysis of the experiment described in (**a**): MyoD and myosin heavy chain (MyHC) expression were checked in differentiated (DM) non-competent AdmMAB clones transduced with retrovirus expressing *wt* MyoD (MyoD), mutated MyoD (MyoDsp3) and empty control vector (Ctl). GAPDH was used to normalize the amount of loaded proteins.

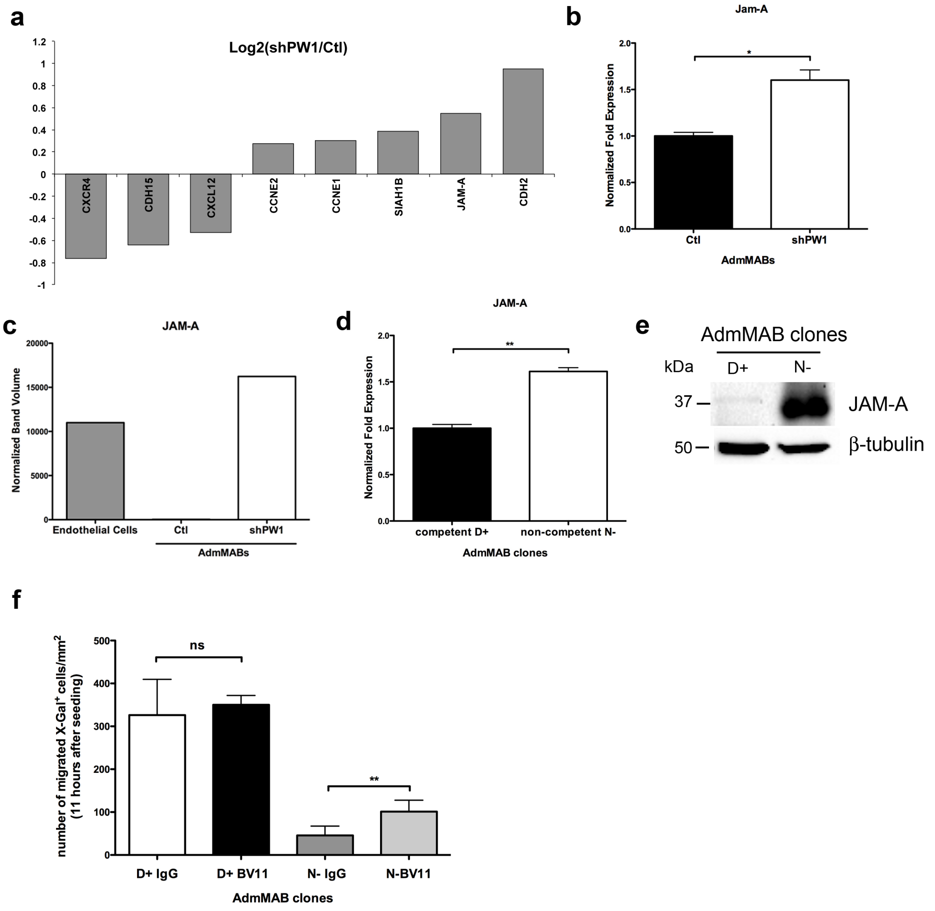

Supplementary Figure 5 JAM-A is up-regulated in mMABs lacking PW1

(**a**) Graphical representation of Affymetrix analysis of the genes upregulated or downregulated in shPW1 AdmMABs *vs* Ctl. Data are expressed as log2 (local-pooled-error test, $p < 0.05$; fold-change >1.2). (**b**) JAM-A expression by qRT-PCR on Ctl and shPW1 AdmMABs. Values are plotted as relative fold expression and normalized to GAPDH levels. Each assay was performed in triplicate. Data are represented as means +/- S.D. *$P < 0.05$, one-way unpaired *t* Test.

(**c**) Quantification of JAM-A expression shown in the Western Blot in Fig.6b on endothelial lung cells, Ctl, shPW1 AdmMABs (ChemiDoc Quantity One 1-D Analysis Software, Biorad).

(**d**) JAM-A expression by qRT-PCR on competent (D+) and non-competent (N-) AdmMAB clones. Values are plotted as relative fold expression and normalized to GAPDH levels. Each assay was performed in triplicate. Data are represented as means +/- S.D. **$P < 0.005$, one-way unpaired *t* Test.

(e) Western Blot analysis of JAM-A expression in competent D+ and non-competent N- AdmMAB clones. β-tubulin was used to normalize the amount of loaded proteins.

(**f**) n-LacZ competent D+ and non-competent N- AdmMAB clones were pre-treated (2 hs) with non-related IgG (20 μg ml^{-1}) and JAM-A neutralizing antibody (anti-JAM-A mAb, 20 μg ml^{-1}, BV11), respectively. Cells were following added to the upper chamber and allowed to migrate for 11 hours. Migrated MABs on the lower side of the filters (X-Gal blue nuclei) were fixed and counted. Quantification of migrated MABs per area is shown. Data are means (±SD) from five independent experiments, each of these was run in triplicate. **$P < 0.005$, ns, not significant, unpaired *t* Test.

Supplementary Western Blots

a

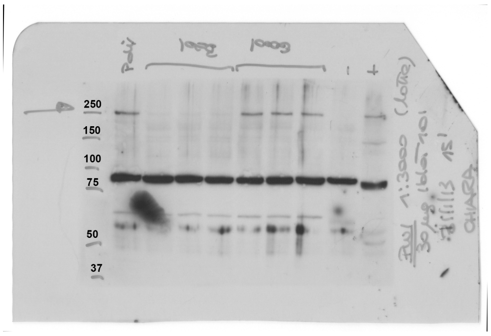

b

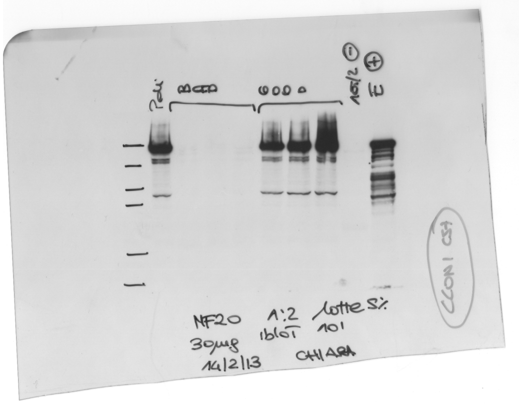

c

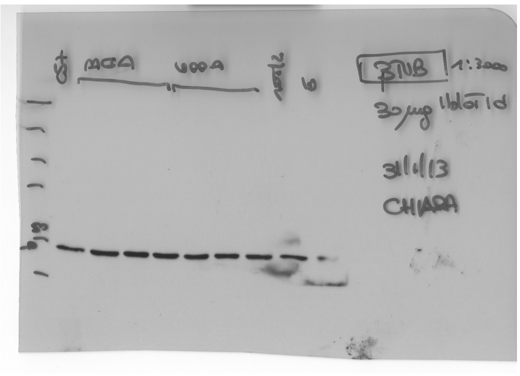

Supplementary Figure 6 Uncropped Western Blots related to the Figure 1c
(a) PW1 (b) MyHC (c) β tubulin.

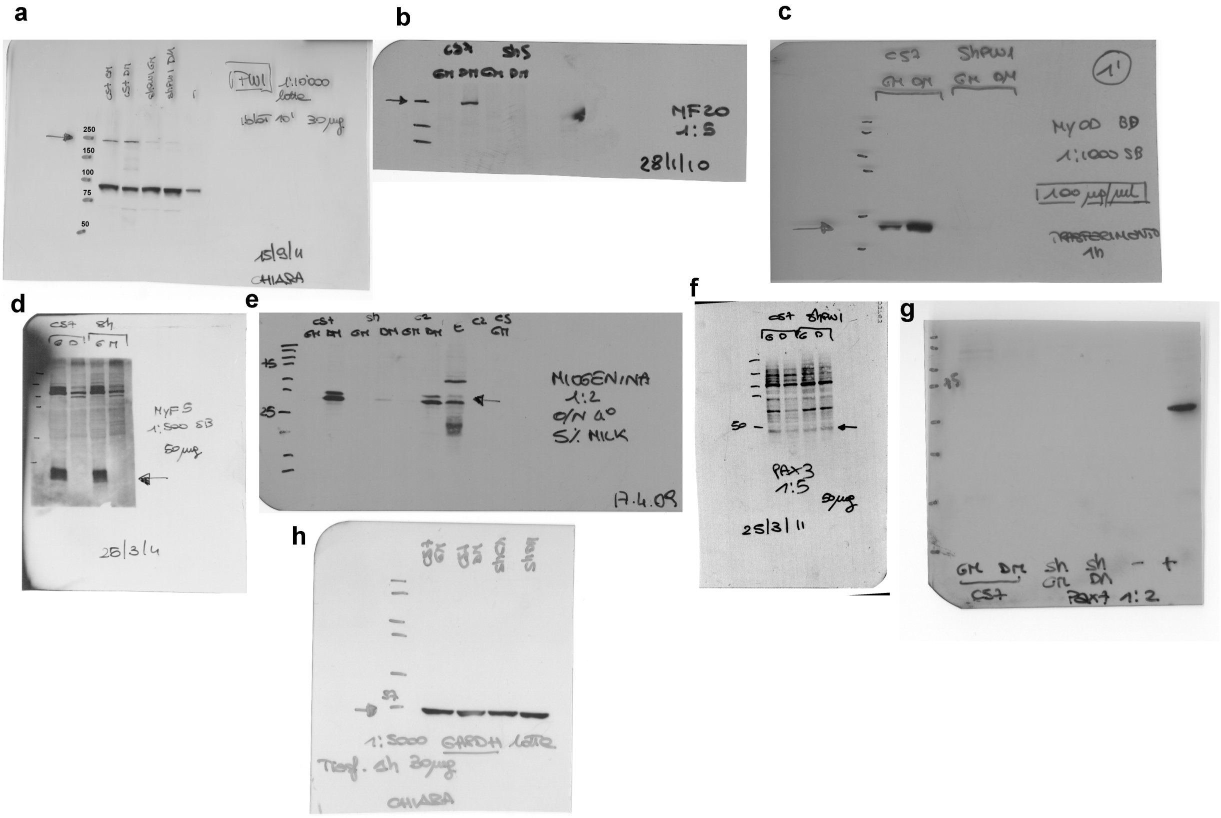

Supplementary Figure 7 Uncropped Western Blots related to the Figure 2a
(a) PW1 (b) MyHC (c) MyoD (d) Myf5 (e) Myogenin (f) Pax3 (g) Pax7 (h) GAPDH.

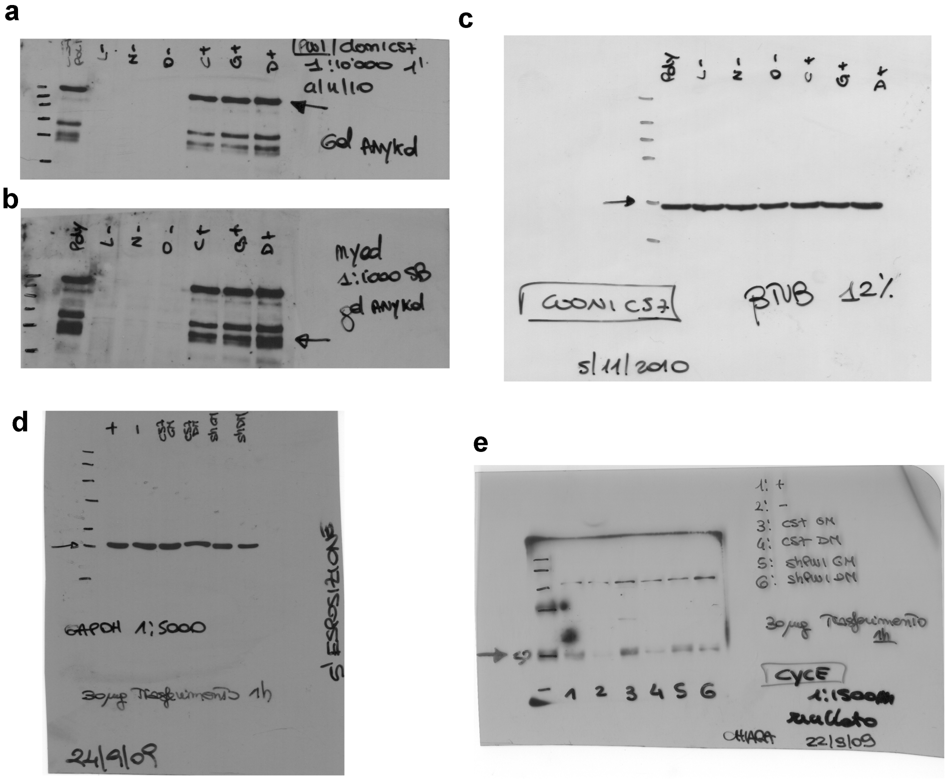

Supplementary Figure 8 Uncropped Western Blots related to the Figure 2c and 2d
(a) PW1 (b) MyoD (c) β-tubulin related to Fig. 2c and (d) GAPDH (e) cyclin E related to Fig.2d.

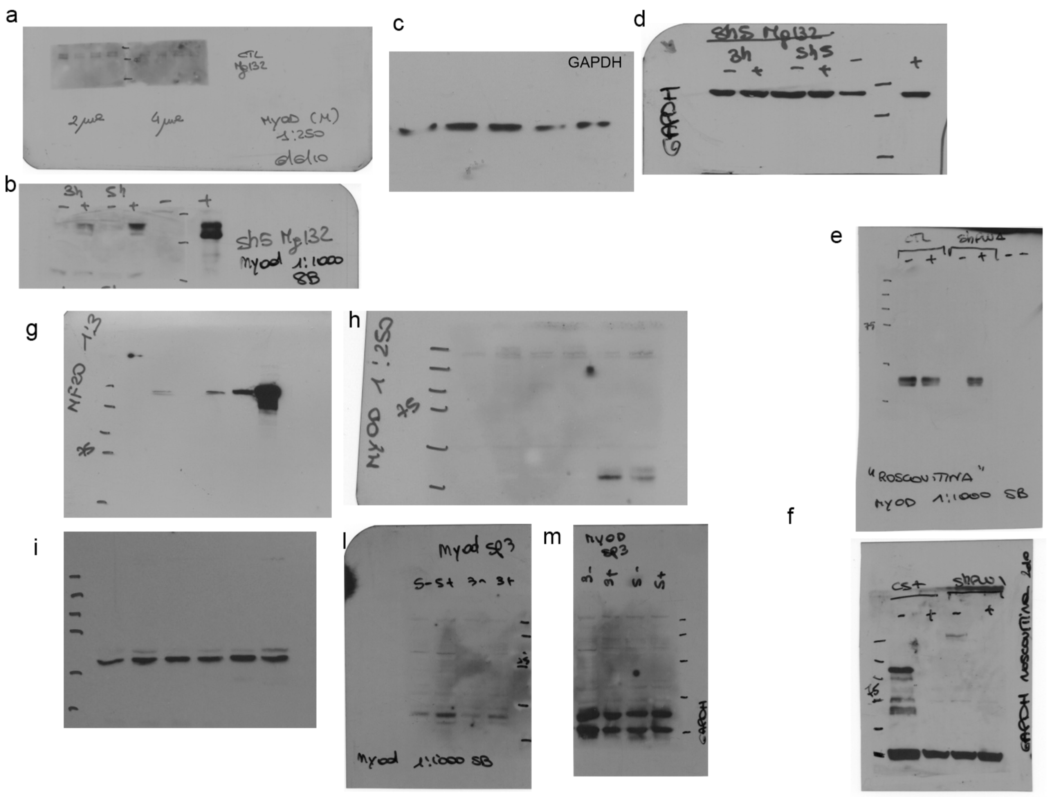

Supplementary Figure 9 Uncropped Western Blots related to the Figure 3a, 3b, 3d and 3e
(a and b) MyoD and (c and d) GAPDH related to Fig.3a, (e) MyoD and (f) GAPDH related to Fig.3b, (g) MyHC (h) MyoD (i) GAPDH related to Fig.3d, (l) MyoD (m) GAPDH related to Fig.3e.

a b c d

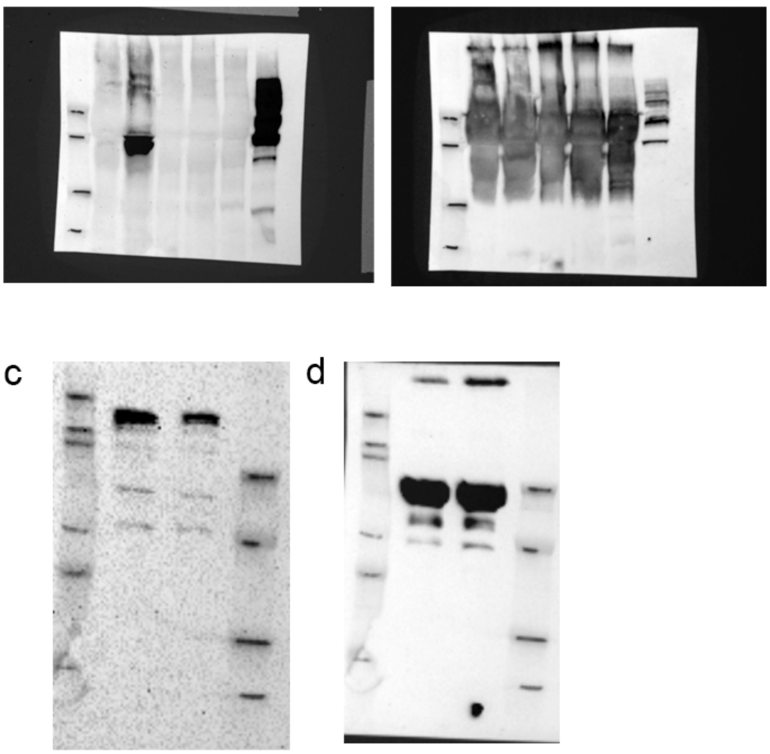

Supplementary Figure 10 Uncropped Western Blots related to the Figure 4 b and 4d
(a) Dystrophin (b) MyHC related to Fig. 4b and (c) Dystrophin (d) MyHC related to Fig.4d.

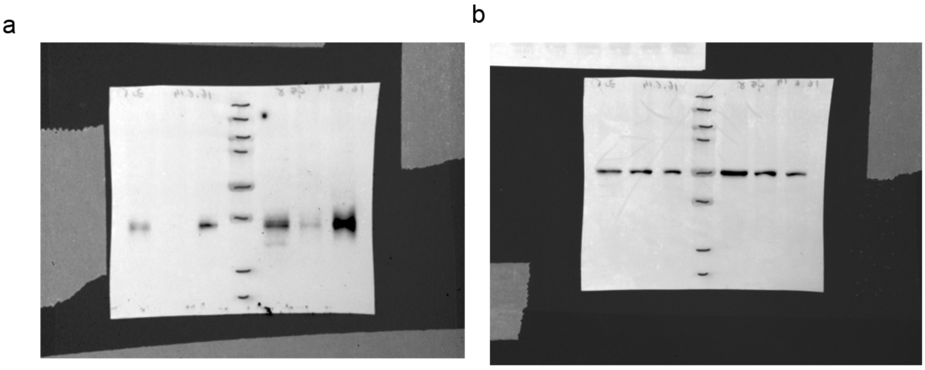

Supplementary Figure 11 Uncropped Western Blots related to the Figure 6b
(a) Jam-A (b) β-tubulin.

Supplementary Table

Supplementary Table 1. Primers used for mRNA expression analysis with quantitative Real Time PCR

Gene	Primers sequence
CycE	F: TTCAGTCCGCTCCAGAAAAAG<br>R: CACAGTCTTGTCAATCTTGGCA
Pw1 (murine):	F: GAGAATCCTCCATTTATATC<br>R: TCATGAATCTTCTGGTGCTC
Pw1 (human):	F: GATCCAAGAGAAGTGCCTACC<br>R: GGAAGATTCATCTTCACAAATCCC
Pw1 (dog)	F: AGCCTCGGCAGGAAGAGCCA<br>R: TCTGCGCGATGTGCAAAGCCT
MyoD	F: ACGGCTCTCTCTGCTCCTTT<br>R: GTAGGGAAGTGTGCGTGCT
JAM-A (murine)	F: TCTCTTCACGTCTATGATCCT<br>R: TTTGATGGACTCGTTCTCGGG
Gapdh	F: AGGTCGGTGTGAACGGATTTG<br>R: TGTAGACCATGTAGTTGAGGTCA

Supplementary Table 2. Primers used for ChiP analysis with quantitative Real Time PCR

Gene	Primers sequence
JAM-A site 1	F: GAGAGCAACCCAGTTTGGGA<br>R: CTGCCTGTGAGATGCAGGAA
JAM-A site 2	F: AGCCACCAATCGGACTCAAG<br>R: CCTCGGTGCCCATTACAGTT
Wnt9a	F: CAGCCATGCTCTAGGGTCACGATA<br>R: GTTTGGCCAGCCTAGTGGGTTT
Intergenic	F: GACCTGCCTGTTCCTTCTTG<br>R: GTTACCCAGCACTGCAAAGG
